# PilG and PilH antagonistically control flagellum-dependent and pili-dependent motility in the phytopathogen *Xanthomonas campestris* pv*. campestris*

**DOI:** 10.1186/s12866-020-1712-3

**Published:** 2020-02-18

**Authors:** Yan-Hua Qi, Li Huang, Guo-Fang Liu, Ming Leng, Guang-Tao Lu

**Affiliations:** State Key Laboratory for Conservation and Utilization of Subtropical Agro-bioresources, College of Life Science and Technology, Guangxi University, 100 Daxue Road, Nanning, 530004 Guangxi China

**Keywords:** Virulence factors, Antagonistic control, Motility, PilG and PilH, *Xanthomonas*

## Abstract

**Background:**

The virulence of the plant pathogen *Xanthomonas campestris* pv*. campestris* (*Xcc*) involves the coordinate expression of many virulence factors, including surface appendages flagellum and type IV pili, which are required for pathogenesis and the colonization of host tissues. Despite many insights gained on the structure and functions played by flagellum and pili in motility, biofilm formation, surface attachment and interactions with bacteriophages, we know little about how these appendages are regulated in *Xcc*.

**Results:**

Here we present evidence demonstrating the role of two single domain response regulators PilG and PilH in the antagonistic control of flagellum-dependent (swimming) and pili-dependent (swarming) motility. Using informative mutagenesis, we reveal PilG positively regulates swimming motility while and negatively regulating swarming motility. Conversely, PilH negatively regulates swimming behaviour while and positively regulating swarming motility. By transcriptome analyses (RNA-seq and RT-PCR) we confirm these observations as PilG is shown to upregulate many genes involved chemotaxis and flagellar biosynthesis but these similar genes were downregulated by PilH. Co-immunoprecipitation, bacterial two-hybrid and pull-down analyses showed that PilH and PilG were able to interact with district subsets of proteins that potentially account for their regulatory impact. Additionally, we present evidence, using mutagenesis that PilG and PilH are involved in other cellular processes, including chemotaxis and virulence.

**Conclusions:**

Taken together, we demonstrate that for the conditions tested PilG and PilH have inverse regulatory effects on flagellum-dependent and pili-dependent motility in *Xcc* and that this regulatory impact depends on these proteins influences on genes/proteins involved in flagellar biosynthesis and pilus assembly.

## Background

*Xanthomonas campestris* is Gram-negative rod-shaped bacteria that causes disease in many plants and is now considered a model organism for the study plant-bacteria interaction [1]. Pathovars of *Xanthomonas campestris* cause many diseases of agronomic importance throughout the world. One of the most notable of these pathogens is *Xanthomonas campestris* pathovar *campestris* (*Xcc*), the causal agent of black rot of crucifers that affects all cultivated brassicas. The diseases caused by *Xcc* are particularly severe in warm and humid regions, although black rot is also known to have a major impact in regions of temperate climate. *Xcc* is also important as a producer of the extracellular polysaccharide (EPS) xanthan, which is used as an additive in the pharmaceutical and food industries.

The virulence of *Xcc* towards plants depends on several pathogenic factors that include extracellular enzymes (such as cellulase, protease, and amylase), EPS, type three effectors and biofilm formation [2–6]. One pathogenic factor of *Xcc* that is gaining more notoriety in virulence is motility. Like most bacteria, *Xcc* uses a variety of extracellular protein structures to interact with their surrounding environment and drives cellular movement. These extracellular protein structures called flagella and pili contribute cellular movement in the form of ‘swimming’ and ‘swarming’, respectively. Additionally, flagellum-dependent and pili-dependent motility are essential to *Xcc*’s ability to attach to host surfaces and to elicit disease. In addition, the flagella and pili are known to be pathogen associated molecular patterns that can induce innate immune responses [7].

Given the importance of flagellum-dependent and pili-dependent motility for *Xcc* survival and ability to cause disease, it is critical that these systems are effectively regulated and controlled. However, despite the numerous studies on bacterial motility in other Gram-negative bacteria, only limited work has been carried out examining the motility regulation in *Xcc*. The majority of these studies describe putative sensor histidine kinases, putative response regulators or cyclic-di-GMP signalling proteins involved in motility regulation which have included RpfG/RpfC [5], RavS/RavR [8], ColR/ColS [9], HpaS/HrpG [10], VemR [11] and VgrR [12]. In majority of cases, deletion or inactivation of the gene encoding these proteins have validated role in flagellum-dependent and pili-dependent motility regulation without much follow up work [1, 12].

Here we present evidence demonstrating the role of previously uncharacterized single domain response regulators (which we designate PilG and PilH) in the regulation of flagellum-dependent and pili-dependent motility in *Xcc*. Using mutagenesis, we show PilG positively regulates swimming motility while and negatively regulating swarming motility. Conversely, PilH negatively regulates swimming behaviour while and positively regulating swarming motility. Our RNA-seq and RT-PCR experiments confirm these observations as PilG is shown to upregulate many genes involved chemotaxis and flagellar biosynthesis but these similar genes were downregulated by PilH. Additionally, we show that PilH and PilG interact with district subsets of proteins using co-immunoprecipitation, bacterial two-hybrid and pull-down analyses. We also present evidence showing that PilG and PilH are involved in other cellular processes. Overall, this analysis reveals that under the conditions tested PilG and PilH are important in the regulation of motility in *Xcc*. Interesting the data shows that both proteins have contrasted regulatory effects on flagellum-dependent and pili-dependent motility which had not been observed previously.

## Results

### PilG and PilH are important for the regulation of pilus-dependent and flagellum-dependent motility in *Xcc*

In our previous work, we isolated large number of *Xcc* mutants from a library constructed using a transposon Tn5*gusA*5 insertion screen of the *Xcc* wild-type strain 8004 (genome accession number CP000050) [13]. Two of these mutants with Tn5*gusA*5 insertions in the open reading frames *XC_1183 and XC_1184* were implicated in motility.

Bioinformatic analysis reveals that the *XC_1183* gene encodes a 133 amino acid protein that shares a high level of identity to the PilG protein from *Pseudomonas aeruginosa*, *Lysobacter enzymogenes* and *Acinetobacter baumannii*, (e value = 5e-77, 8e-84 and 8e-74, respectively) (Additional file 1: Figure S1A). Interestingly, the *XC_1184* gene also encoded a 120 amino acid protein that had 50% (e value = 4e-45) amino acid homology with PilH from *P. aeruginosa*, 72% (e value = 3e-66) amino acid homology with PilH from *L. enzymogenes*, and 52% (e value = 2e-46) amino acid homology with PilH from *A. baumannii*, respectively (Additional file 1: Figure S1B). We designated the proteins XC_1183 and XC_1184 in *Xcc* as PilG and PilH for the remainder of the study. Domain analysis using the SMART (Simple Modular Architecture Research Tool) programme (http://smart.embl-heidelberg.de) showed that PilG (XC_1183) and PilH (XC_1184) both contained a stand-alone CheY-like REC domain (PilG: 14aa-127aa, PilH: 2aa-115aa) (Additional file 1: Figure S1C). Further comparison of PilG and PilH to the well-studied CheY protein domain of *Escherichia coli* was also carried out*.* These comparisons showed that PilG shared a 29% (e value = 2e-20) amino acid homology with CheY, while PilH showed a 30% (e value = 3e-16) amino acid homology (Additional file 1: Figure S1C). Additionally, the previously characterized CheY protein from *Xcc* revealed a 39% (e value = 6e-27) amino acid homology with its *E. coli* counterpart (Additional file 1: Figure S1C).

PilG and PilH have been shown to be required for motility in many bacteria, including *P. aeruginosa* [14, 15] and *Neisseria meningitidis* [16] but no roles have been attributed to these proteins in *Xcc*. In order to explore the function of PilG and PilH in *Xcc*, we constructed clean deletions removing *XC_1183* and *XC_1184* gene and designating the resulting strains as ΔpilG and ΔpilH (see Methods; Additional file 6: Table S1). Simultaneously, these strains were complemented by introducing the plasmid pLAFR3 carrying the *XC_1183* or *XC_1184* coding sequence along into the respective mutant. The resulting complemented strains were named CpilG and CpilH (Additional file 6: Table S1).

**Fig. 1:**
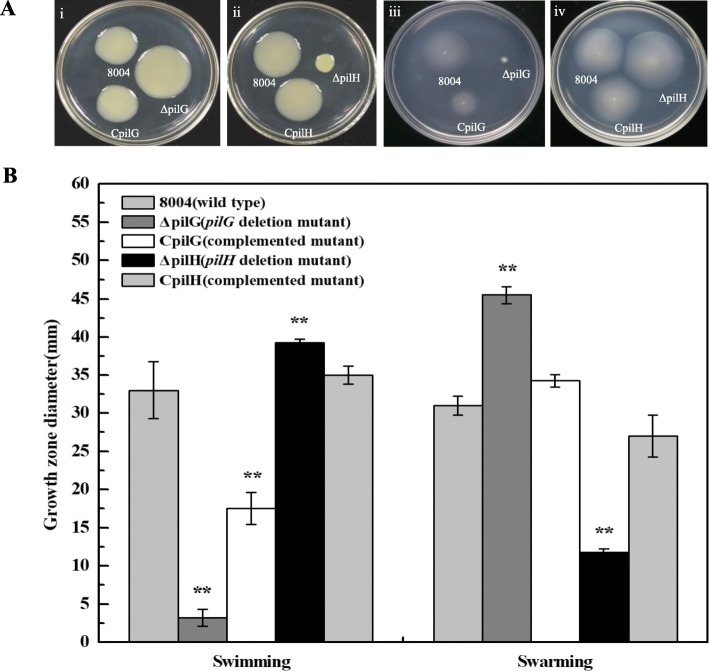
PilG and PilH antagonistically regulate swimming and swarming motility in *Xcc*. **a** Examination of swimming and swarming motility. i and ii Examination of swarming motility for mutation in genes *pilG* and *pilH*. Strains were inoculated onto ‘swarm’ plate (NY medium containing 2% glucose and 0.6% agar) then incubated at 28 °C for 3 days. iii and iv Examination of swimming motility for mutation in genes *pilG* and *pilH*. Strains were stabbed into ‘swim’ plate (0.03% Bacto peptone, 0.03% yeast extract and 0.28% agar) then incubated at 28 °C for 3 days. **b** The diameter of the colony 8004, ΔpilG, CpilG, ΔpilH and CpilH on swimming and swarming plates. Significance was tested by Student’s t test (* and ** represent significance at *P* < 0.05 and 0.01, respectively)

Pilus-dependent swarming motility has previously been shown contribute to disease in *Xcc* [17]. To determine if PilG and PilH are involved in pilus-dependent swarming motility, the constructed strains and wild-type were tested by inoculating on NY plates containing 2% glucose and 0.6% agar then incubating at 28 °C for 3 days (see Methods) [18]. As shown in Fig. 1, ΔpilG displayed increase 40 % in net migration compared to the wild-type, while showed a 60 % decrease in motility compared to the wild-type (Fig. 1). Importantly, the complementary strains CpilG and CpilH showed similar motility phenotypes to wild-type (Fig. 1). These findings suggest that PilG negative regulates pilus-dependent swarming in *Xcc*, while PilH appears to act as a positive regulator.

*Xcc* has also the ability to swim using its single polar flagellum [19]. To investigate if PilG and PilH are involved in flagellum-dependent swimming motility, the mutants ΔpilG and ΔpilH, complemented strains CpilG and CpilH, and wild-type strain were inoculated onto 0.28% agar plates and incubated for 3 days (see Methods) [18]. As shown in Fig. 1, ΔpilG showed reduced 90 % compared to wild-type. However, the ΔpilH deletion strain showed increase of 20 % in swimming motility compared to the wild-type. Importantly, swimming motility of the complementary strains CpilG could be back to half of the wild type and CpilH showed the restoration of swimming motility phenotypes towards wild-type (Fig. 1). This data suggests that PilG is a positive regulator of flagellum-based swimming but PilH appears to act as a negative regulator which is the converse of what was observed in the pilus-based swarming results.

Taken together, the mutagenesis data described above revealed that PilG and PilH have opposite roles for the regulation of pilus-dependent swarming and flagellum-dependent swimming in *Xcc*.

### PilG and PilH influence chemotaxis and virulence in *Xcc*

To explore if PilG and PilH manipulate additional specific functions that are known to be associated with motility and virulence, we conducted a series of phenotypic tests to assess growth, EPS, chemotaxis and virulence in *Xcc* (see Methods).

**Fig. 2:**
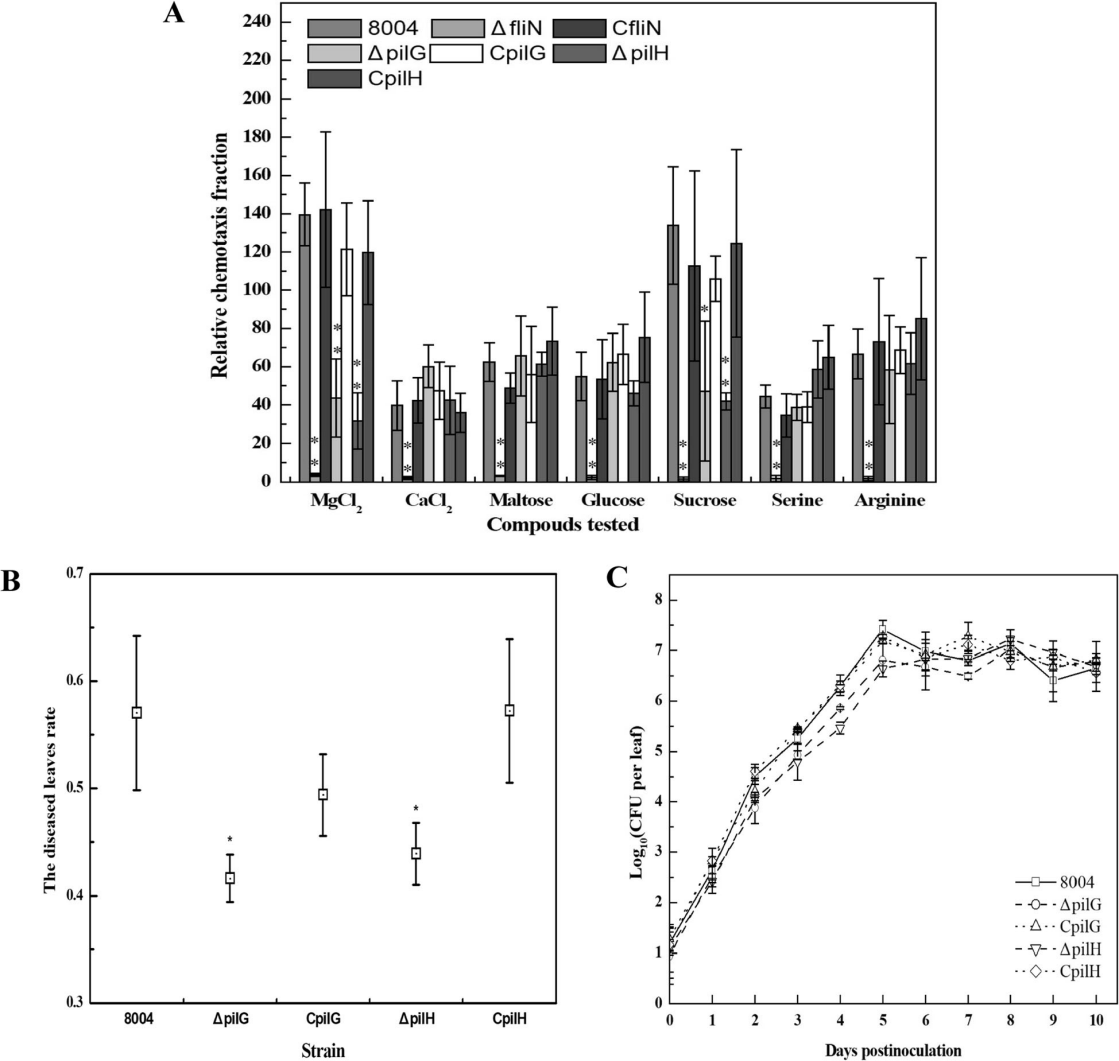
PilG and PilH influence diverse cellular processes including chemotaxis and virulence*.***a** Chemotaxis response assay. The strains were diluted and performed simplified capillary assays for 8 chemoattractant and counted bacterial CFUs then incubating at 28 °C for 3 days. Relative chemotaxis fraction was calculated CFU in test capillary vs CFU in control buffer capillary. **b** The virulence of *Xcc* strains. The strains were diluted and sprayed onto leaves of 100 radishes showing similar growth, and counted diseased leaves rate after 10 days. Additionally, each strain was repeated three times. **c** In-plant growth curve. The strains were diluted and inoculated to Chinese radish by leaf-clipping. At intervals of 1 day, four clipped leaves for every group of inoculated plants were collected and homogenized, and then diluted to plate on NYG plates. The colonies were counted after 3 days of incubation at 28 °C. Significance was tested by Student’s t test (* and ** represent significance at *P* < 0.05 and 0.01, respectively)

As shown in Additional file 2: Figure S2, production of EPS in ΔpilG was similarity to the wild type but ΔpilH decreased production of EPS greatly, and then no influence on growth between mutants and wild type in NY medium. Additionally, differences were seen when the wild-type strain, the ΔpilG and ΔpilH mutants and the complemented strains ability to sense and respond chemotactic agents was assessed. For these experiments, a simplified capillary assay for the qualitative analysis of chemotaxis was used (see Methods). Here eight selected chemoattractant (two inorganic salts [CaCl_2_, MgCl_2_], three carbohydrates [maltose, glucose, sucrose] and two amino acid [serine, arginine]) and a negative control (H_2_O) were used to assess the chemotactic response of *Xcc*. The results revealed that ΔpilG and ΔpilH strains exhibited a significantly lower response to MgCl_2_ and sucrose compared with wild-type (Fig. 2a). However, no differences between complementary strains CpilG and CpilH and wild-type were seen. These results indicated that PilG and PilH have no effect on growth in NY medium and regulate the chemotactic response of *Xcc*.

Interestingly, alterations were seen when strains were assessed for their virulence. The results showed that the ΔpilG and ΔpilH mutants displayed decreased diseased leaves rate via spraying inoculation onto radish leaves (Fig. 2b), but the virulence was no differences for ΔpilG and ΔpilH mutants though leaf-clipping to infect radish leaves (data not shown). To determine if growth in planta contributes to plant pathogenicity, we determined the growth of strains. As shown in Fig. 2c, ΔpilG and ΔpilH have slower growth in planta compared to wild-type and the corresponding complemented strains. These data indicated that PilG and PilH have effects on pathogenicity mainly to be colonized and grown on host tissues.

Taken together, these findings suggested that PilG and PilH contribute to the regulation of chemotaxis and virulence in *Xcc* under the conditions tested. Despite these observations, the mechanism of regulation of chemotaxis and virulence by PilG and PilH remains to be further understood.

### PilG and PilH have an influence over the expression of genes involved in chemotaxis, flagellar biosynthesis and pilus assembly in *Xcc*

The findings outlined above showed that PilG and PilH play complex roles in the regulation of pilus-dependent and flagellum-dependent motility and other associated phenotypes. To gain a greater understanding of the regulatory role of PilG and PilH in *Xcc* a set of global gene expression profiles were generated using RNA-seq and RT-PCR. For these experiments, total RNA was purified from the wild-type, ΔpilG and ΔpilH strains grown to the mid-exponential phase (OD_600_ = 1.0) in NYG medium, and tested quantity and quality (see Methods).

**Fig. 3:**
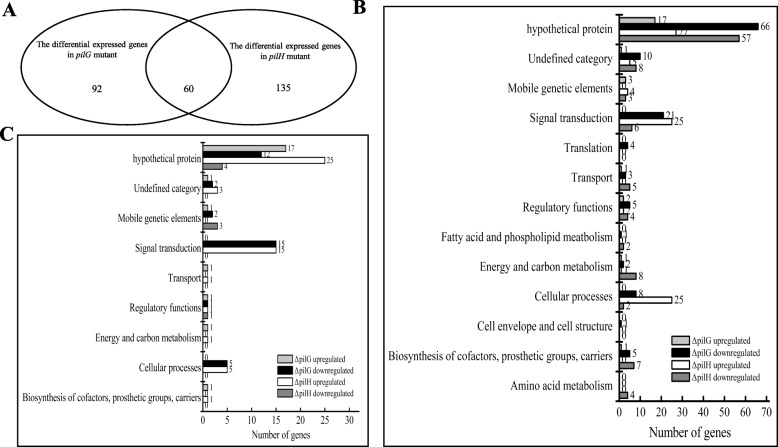
Functional categories of the differential expressed genes in two mutant backgrounds. **a** The venn diagram showed the overlap between the differential expressed genes in two mutant backgrounds. **b** Functional categories of the differential expressed genes in two mutant backgrounds. The differential expressed genes from Additional file 7: Table S2 and Additional file 8: Table S3 were broadly categorized according to their biological function. **c** Functional categories of overlap of the differential expressed genes in two mutant backgrounds. The differential expressed genes from Additional file 9: Table S4 were broadly categorized according to their biological function. Each bar represents the number of differential expressed genes in each category of *Xcc* 8004 genome

Following bacterial RNA extraction and sequencing, differential gene expression analysis was conducted on the generated data (see Methods). A false discovery rate of FDR ≤ 0.05, and |log_2_FC| (log_2_ of the fold changes) ≥1 was considered for differentially expressed genes. Comparison of transcriptome data between the wild-type, ΔpilG and ΔpilH strains reveal significant differences. In the absence of PilG a total of 152 genes were altered in which 125 genes were down-regulated and 27 genes were up-regulated (Additional file 7: Table S2). However, in the absence of PilH a total of 195 genes revealed significantly altered expression in which 106 were down-regulated and 89 were up-regulated genes (Additional file 8: Table S3). Interestingly, only 60 expressed genes appear to co-regulated by PilG and PilH (Fig. 3a). To validate the transcriptome data and confirm the changes in the co-regulated genes a total of 18 genes were selected and the expression changes were validated by semi-quantitative RT-PCR (Additional file 3: Figure S3). These results confirmed that the differential gene expression analyses based on our transcriptome are reliable.

The genes that showed expression changes were subjected to functional categorization using KEGG using the *Xcc* 8004 genome annotation [20]. Based on biological functions analysis, all of the genes that changed were mainly catalogued into 13 functional categories that included signal transduction, biosynthesis and metabolism (Fig. 3b). Despite genes being ascribed to a broad number of functional categories majority of regulated genes were predicted to encode hypothetical proteins or did not have a functional category assigned to date. This included 85 of 152 genes regulated by PilG and 84 of 195 genes regulated by PilH. While 29 of the 60 genes co-regulated by both PilG and PilH were predicted to encode hypothetical proteins (Fig. 3c).

Interestingly, examination of the remaining co-regulated genes showed they were associated with functions relating to motility, specifically chemotaxis, flagellum and pilus assembly and function (Additional file 9: Table S4). Consistent with the motility phenotypes attributed to be under the control of PilG and PilH it was clear that these genes were divergently regulated (Fig. 3c). For example, 16 chemotaxis-associated genes (*XC_2320*, *XC_2302*, *XC_2311*, *XC_2309*, *XC_2223*, *XC_1413*, *XC_0638*, *XC_2321*, *XC_2306*, *XC_2318*, *XC_1410*, *XC_2303*, *XC_2314*, *XC_1414*, *XC_2313*, *XC_2298*), and 4 flagellum-related genes (*XC_2245*, *XC_2264*, *XC_2231*, *XC_2246*) were found to be down-regulated in the ΔpilG mutant, but up-regulated in the ΔpilH mutant (Additional file 9: Table S4). Furthermore, *pilI* (*XC_1185*) and *pilJ* (*XC_1186*) involved in pilus-dependent motility were down-regulated in the ΔpilH strain but were unchanged in the ΔpilG strain.

Taken this data together it is clear the impact of PilG and PilH on the expression of specific genes at the transcriptional level accounts for the phenotypes seen in the ΔpilG and ΔpilH strains. Furthermore, these results indicate PilG and PilH control pilus-dependent and flagellum-based motility by potentially opposingly regulating genes involved in flagellar biosynthesis and pilus assembly.

### Mutation in genes that encoding structural elements of the pili, flagellum and chemotaxis systems reveal similar phenotypes to *pilG* and *pilH* in *Xcc*

The gene clusters encoding proteins needed to generate the flagellar and pili systems in *Xcc* have previously been examined in strains Xc17 and ATCC 33913 [19]. In the strain used in the current study was examined for the presence of flagellar and pili genes along with known regulatory genes. By these analyses, we found the presence of one cluster of motility-related (flagellar and chemotaxis) genes, and several pili-related genes. In order to examine the impact of these genes on the phenotypes that appear to be controlled by PilG and PilH, the effects of mutation of specific genes were assessed (see Methods). Specifically, we constructed in-frame deletion mutations in flagellum-assembled genes *fliM* and *fliN* (*XC_2267* and *XC_2268*), a pili-assembled gene *pilB* (*XC_1060*) and chemotaxis-associated genes (*XC_1414*, *XC_2302* and *XC_2306*) (see Methods). These strains were designated ΔfliM, ΔfliN, ΔpilB, ΔcheA, ΔcheY and Δ2306, respectively (Additional file 6: Table S1). In tandem, these mutants were complemented and the resulting strains were named CfliM, CfliN, CpilB, CcheA, CcheY and C2306 (Additional file 6: Table S1). This functional genomic examination of those structural elements of the pili, flagellum and chemotaxis systems that may indirectly contribute to phenotypes of *pilG* and *pilH* mutants.

**Fig. 4:**
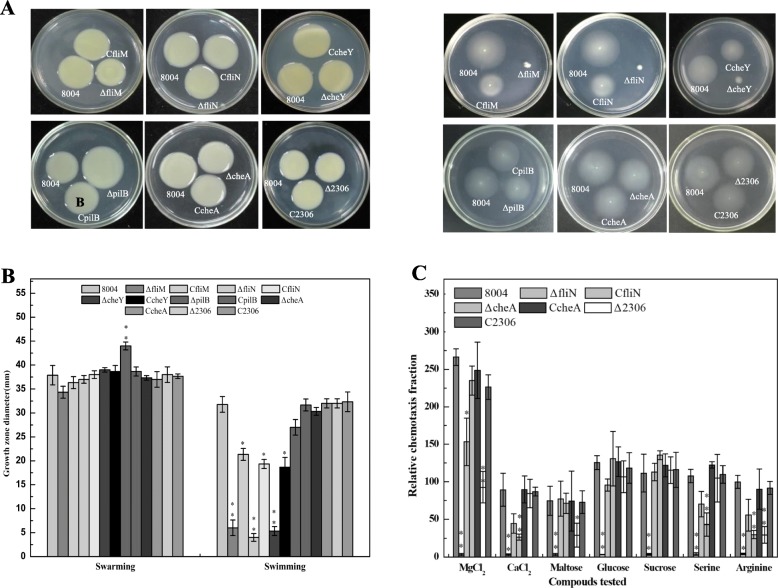
Phenotypic characterization of mutation in six motility-related (pili, flagellar and chemotaxis) genes. **a** Examination of swimming and swarming motility. Left side, strains were inoculated onto ‘swarm’ plate (NY medium containing 2% glucose and 0.6% agar) then incubated at 28 °C for 3 days. Right side, strains were stabbed into ‘swim’ plate (0.03% Bacto peptone, 0.03% yeast extract and 0.28% agar) then incubated at 28 °C for 3 days. **b** The diameter of the strains on swimming and swarming plates. **c** Chemotaxis response assay. The strains were diluted and performed simplified capillary assays for 8 chemoattractant, and counted bacterial CFUs after 3 days of incubation at 28 °C. Relative chemotaxis fraction was calculated CFU in test capillary vs CFU in control buffer capillary. All datas were representative of three independent experiments. Significance was tested by Student’s t test (* and ** represent significance at *P* < 0.05 and 0.01, respectively)

The pilus-dependent and flagellum-based motility of each mutant were tested (see Methods). Mutation of *fliM* (*XC_2267*), *fliN* (*XC_2268*) and *cheY* (*XC_2302*) led 80 % loss of flagellum-based swimming compared to the wild-type (Fig. 4a and b) and their corresponding complementary strains could be back to 60 % of the wild type in phenotype. Interestingly, mutation of *pilB* led to an increase of 20 % approximately in swarming compared to the wild-type (Fig. 4a and b), and then complementation restored the phenotypes towards wild-type (Fig. 4a and b). None of the other mutants showed any significant difference in motility compared to wild-type.

In addition, alterations were seen when strains were assessed for their response to chemotactic agents (see Methods). The two chemotaxis-associated genes (*XC_1414* and *XC_2306*) were found to exhibit a significant difference in chemotaxis response. Analysis showed that there were significant differences in ΔcheA (*XC_1414*) compared to wild-type toward CaCl_2_, serine and arginine (Fig. 4c). While the Δ2306 (*XC_2306*) showed a difference in movement toward MgCl_2_, maltose and arginine compared to wild-type (Fig. 4c).

### PilG and PilH interact directly with district subsets of proteins that potentially account for their regulatory role

The majolity of CheY-like proteins (contain a stand-alone REC domain) have been shown to regulate motility by intermolecular interactions with motor proteins. While other stand-alone REC domains, such as *Bacillus subtilis* Spo0F, have been shown to function as phosphorylated intermediate in phosphorelay pathways [21–24]. The observations discussed above indicate that PilG and PilH regulate the expression of genes that indirectly influence *Xcc* motility but also that a set of structural elements of the pili, flagellum and chemotaxis systems that may contribute to phenotypes of *pilG* and *pilH* mutants.

**Fig. 5:**
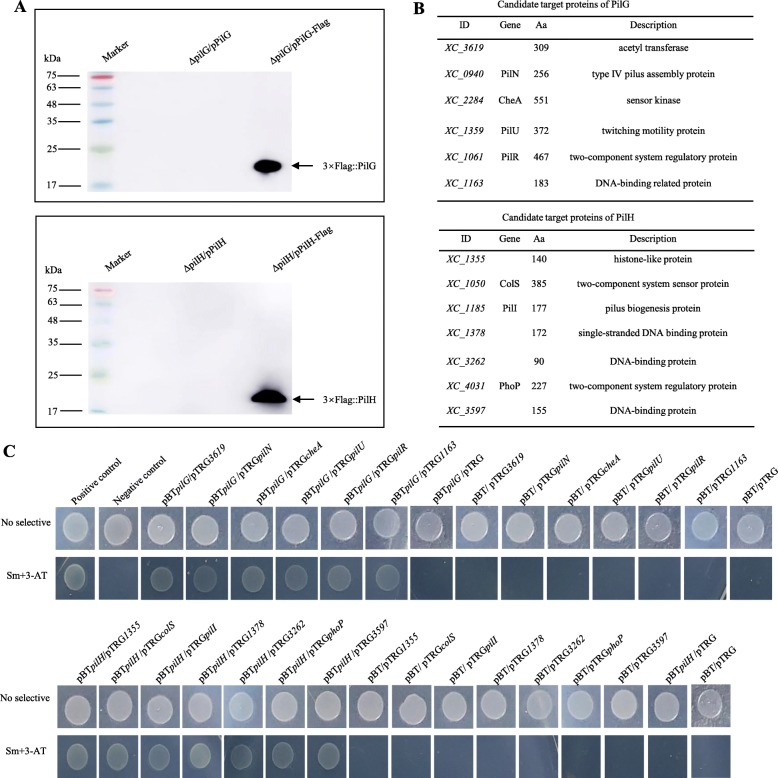
Identification of proteins that interact with PilG and PilH in *Xcc*. **a** Western blotting of the eluted 3 × Flag::PilG fusion protein and the eluted 3 × Flag::PilH fusion protein. After Co-immunoprecipitation, a western blot assay was performed for the eluted 3 × Flag::PilG (or 3 × Flag::PilH)fusion protein and the control. **b** Candidate target proteins of PilG and PilH from two MS-coupled co-IP experiments. **c** The bacterial two-hybrid experiment showed that PilG and PilH interacted with candidate target proteins. The reporter strain XL1-Blue MRF′ with different plasmid pairs was grown on no selective plates and double-selective indicator plates containing 5 mM 3-AT and 12.5 μg ml^− 1^ streptomycin. Protein-protein interactions activated the expression of *addA* and *HIS3* genes within the reporter gene cassette of the reporter strain, resulting in resistance to streptomycin and 3-AT

To examine if PilG and PilH potentially interact specifically with proteins, we initially employed co-immunoprecipitation (co-IP) coupled with and mass spectroscopy (see Methods). For these experiments a 3 × Flag-tagged fusion protein 3 × Flag::PilG (or PilH) was constructed (Additional file 6: Table S1), where a 3 × Flag-tag was fused to the 5’end of the *pilG* gene (or *pilH* gene) and cloned into the vector pLAFR3. These constructs were introduced into strains of interest including the respective mutant (ΔpilG or ΔpilH), and the complemented strain (CpilG or CpilH) for not expressing 3 × Flag-tagged fusion proteins as a negative control (Additional file 6: Table S1). A western blot assay confirmed that the 3 × Flag::PilG (or PilH) fusion protein complex could be eluted from strains for expressing 3 × Flag-tagged fusion protein, but not the control strain CpilG (or CpilH) for not expressing 3 × Flag-tagged fusion proteins (Fig. 5a). The MS-coupled co-IP experiment was repeated twice and take the same results but removing results from negative control as the candidate target proteins. The 3 × Flag::PilG (or PilH) fusion protein complexes purified from the ΔpilG mutant the identities of 6 interacting proteins could be established by mass spectrometry (Fig. 5b). Interestingly, the 3 × Flag::PilH fusion identified a completely different set of 7 interacting proteins (Fig. 5b). These proteins were shown to have roles in signalling (sensor kinases), regulation (DNA-binding regulators) and importantly motility.

The co-IP analysis was extended and validated by using a bacterial two hybrid and biotinylated protein-protein blotting experiments. Importantly, the PilG and PilH protein were shown to interact with their respective proteins using bacterial two hybrid assay (Fig. 5c). PilG confirmed directly interaction with XC_0940, XC_1061, XC_1163, XC_1359, XC_2284 and XC_3619. When PilH was tested it showed interactions with XC_1050, XC_3262, XC_1185, XC_1355, XC_1378, XC_3597 and XC_4031. Interestingly, both PilG and PilH proteins interacted with FilN by using bacterial two hybrid and biotinylated protein-protein blotting experiments (Additional file 4: Figure S4). All interactions were detected for a second time. Taken together, these data suggested that PilG and PilH interact with two distinct subsets of proteins that have roles in signalling, regulation and importantly motility.

### Functional genomic assessment of genes encoding protein that interact with PilG and PilH identifies a novel regulator involved in *Xcc* motility

As mutation of *pilG* and *pilH* lead to an alteration in motility and associated traits in *Xcc*, it follows that mutation of genes that encode proteins that interact with these regulators may have a role in motility. This was confirmed by our previous work which has shown that mutation of genes encoding PilN (XC_0940), PilU (XC_1359), PilR (XC_1061), XC_3262 and PhoP (XC_4031) have been shown to play a role *Xcc* motility [17, 25–27].

In order to examine this the other proteins that interacted with PilH and PilG, the effects of mutation on selected genes were assessed swimming and swarming motility (see Methods). The strain carrying a deletion in *XC_1185* demonstrated the most profound effect on swarming motility (Additional file 5: Figure S5). *XC_1185* encodes a signalling protein containing a CheW domain and homologs of this protein are named PilI in strains of *Pseudomonas* sp. Importantly, swarming motility was restored towards wild-type by complementation *in trans* with a wild-type copy (Additional file 5: Figure S5).

Taken together, these findings suggested that proteins that interact with PilG and PilH may regulate motility in a similar indirection under the conditions tested. Furthermore, a new factor PilI (*XC_1185*) which interacts with PilH directs has partly the same phenotypes. Interestingly, this protein has homologues in other plant associated bacteria, including other *Xanthomonas* species. However, despite these observations, the mechanism of regulation by PilG and PilH in these cases remains enigmatic and the question of how PilG, and in particular PilH, influences these proteins regulatory roles is yet to be understood.

## Discussion

The surface appendages flagellum and pili are the most commonly studied motility devices in bacteria. Polar flagella are known to act as ‘helical propellers’, whereas the pili act as ‘grapnel’ or ‘anchors’ [28]. These systems can also contribute to biofilm formation, surface attachment and chemotaxis. In many bacteria, PilG and PilH have been shown to regulate pilus-dependent motility [14, 15, 29]. In the current study, we present evidence demonstrating the roles of PilG and PilH in the regulation of motility of *Xcc* are more complex than previously described in other Gram-negative bacteria. The data consistent with the hypothesis that PilG and PilH have antagonistic regulatory effects on flagellum-dependent and pili-dependent motility, where PilG and PilH appear to exert their regulatory influence by interacting directly with a subset of proteins involved in flagellar biosynthesis and pilus assembly or controlling gene expression.

Through the construction and complementation of deletion mutants, we revealed that PilG positively regulates flagellum-dependent motility while and negatively regulating pili-dependent motility. Conversely, PilH was shown to negatively regulate flagellum-dependent behaviour while and positively regulating pili-dependent motility. In addition to motility, little is known about specific functions of single-domain response regulators [30], so we were looking for functional information of PilG and PilH though transcriptional regulation and protein-protein interaction mediated regulation. These observations were supported by transcriptome analyses that showed PilG up-regulating many genes involved chemotaxis and flagellar biosynthesis but these genes were inversely regulated by PilH. It is clear that PilG and PilH appear to play unique roles in controlling motility in *Xcc*. A better understanding of how PilG and PilH might influence motility was revealed by the MS-coupled co-IP experiments. We found that PilG could interact with PilN, PilR, PilU, CheA, XC_3619 and XC_1163. While PilH interacted with PilI, ColS, PhoP, XC_1355, XC_1378, XC_3262 and XC_3597. Importantly, our previous work has shown that mutation of genes encoding PilN (XC_0940), PilU (XC_1359), PilR (XC_1061), XC_3262 and PhoP (XC_4031) have been shown to play a role *Xcc* motility [17, 25–27]. Furthermore, additional mutagenesis revealed that a new factor PilI (XC_1185) which interacts with PilH has partly the same phenotypes in *Xcc*.

It has been demonstrated in *P. aeruginosa* that PilH directly influences PilI and PilJ which in turn controls the production of PilA [15]. Therefore, this might be one reason why PilH in *Xcc* may regulate pili-dependent motility as it interacts with PilI. Nonetheless, the way PilG independently regulates pili-dependent motility is not clear. The three pili related proteins that PilG interacts with are PilN, a pili-assembly protein, which may form an inner membrane subcomplex with PilO and PilP to influence alignment of the secretin for the pilus assembly machinery [31]; PilR, shown to function as a transcriptional activator of biosynthesis of PilA in *Xanthomonas axonopodis* pv. *citri* [32] and PilU, a ATPase able to provide energy for extension or contraction of pili [26, 33]. However, the RNA-Seq data revealed that PilG did not affect the expression of PilA, indicating that PilG might regulate swarming via interacting with PilN and PilU to affect assembly. Further work is required to confirm this.

Although PilG and PilH appear to interact directly with subsets of proteins, the influence on these proteins is still very much in question. Many proteins containing stand-alone REC domains, such as *B. subtilis* Spo0F, have been shown to function as phosphorylated intermediate in phosphorelay pathways [21–24]. Maybe PilG and PilH accept phosphoryl groups from the sensor kinase CheA and ColS, respectively. However, PilG and PilH accepting phosphoryl groups from CheA and ColS provides clues to how this protein is involved in regulation of flagellar motility. It has been demonstrated in *E.coli* that the direction of flagellar rotation is regulated via phosphorylated CheY bound to the N-terminal segment of FliM [21–24], and that the C-terminal segment of FliM is flexible enough to allow subsequent binding to a site on FliN in the vicinity of the hydrophobic patch [34], finally it destabilizes the interaction between FliM and the C-terminus of FliG and FliG is bound to MotA that causes the flagellar rotation [35, 36]. However, a more recent work has found that chemotaxis signalling protein CheY binds to the rotor protein FliN to control the direction of flagellar rotation in *E coli* [37]. Here a direct interaction between PilG and flagellar proteins FliN was detected based on bacterial two-hybrid assay and pull-down biotinylated protein-protein assay, suggesting that PilG may influence flagellum-dependent motility though interaction with FliN. However, the role of PilH in flagellum-dependent motility remains enigmatic. Work in *P. aeruginosa* has suggested that PilH may not directly regulate flagellum-based motility but act as a phosphate sink [38]. This remains to be confirmed in *Xcc*.

In addition to the influence over flagellum-dependent and pili-dependent motility, *pilG* and *pilH* deletion mutants showed decreased virulence and chemotaxis ability for MgCl_2_ and sucrose, suggesting that these signalling protein interfaces with multiple signal transduction circuits in *Xcc*. Transcriptome analysis revealed that the chemotaxis-associated genes were downregulated in *pilG* mutant and upregulated in *pilH* mutant, indicating that *pilG* and *pilH* may promote chemotaxis ability of *Xcc* though altering expression of chemotaxis-associated genes. Despite these observations, the mechanism of regulation of virulence and chemotaxis by PilG and PilH remains to be further understood.

In summary, our study has identified that PilG and PilH as two multifunctional regulators that control diverse cellular processes including swarming, swimming, surface adherence and chemotaxis in *Xcc*. Specifically, the influence of PilG and PilH on motility in *Xcc* is likely the result of two fold where: (1) PilG and PilH antagonistically affect the expression of genes involved in flagellum and pili-dependent motility; (2) PilG and PilH interact with subsets of proteins that are involved in pili-dependent motility. Despite these advances further studies are needed to examine the role of PilG and PilH in the regulation of the additional phenotypes they have been shown to regulate but also suggest several other questions in to be addressed: What are the environmental cues that activate the expression and activity of PilG and PilH? Does PilG and PilH regulate differently during plant colonization? How does PilG and PilH directly influence the proetins they interact with? How does PilG and PilH affect gene expression in? Is PilG and PilH involved in phosphotransfer? Do PilG and PilH have conserved protein interaction sites?

## Conclusions

In this work, we found that mutation of the gene encoding PilG and PilH antagonistically control flagellum-dependent and pili-dependent motility, and present evidence showing that PilG and PilH are involved in other cellular processes. In summary, we demonstrate that (1) PilG and PilH antagonistically affect the expression of genes involved in flagellum and pili-dependent motility; (2) PilG and PilH interact with subsets of proteins that are involved in pili-dependent motility.

## Methods

### Bacterial strains and culture conditions

The bacterial strains and plasmids used in this study were listed in Additional file 6: Table S1. *Escherichia coli* strains were routinely grown in Luria-Bertani broth at 37 °C. *Xcc* strains were grown at 28 °C in NYG medium (peptone, 5 gL^− 1^; yeast extract,3 gL^− 1^; and glycerol, 20 gL^− 1^, pH 7.0) [39]. Antibiotics were added at the following concentrations as required: kanamycin (Kan) 25μgml^− 1^, rifampicin (Rif) 50μgml^− 1^ and tetracycline (Tet) 5μgml^−1^for *Xanthomonas spp*. and 15 μgml^−1^for *E. coli*.

### Construction of in-frame deletion mutants and its genomic integrated complemented strains

In-frame deletion mutant was constructed by two exchange steps using the plasmid pK18mobsacB [40]. For construction of *Xcc pilG* deletion mutant, 521-bp upstream and 327-bp downstream fragments flanking *pilG* (*XC_1183*) were amplified using the total DNA of the *Xcc* wild type strain 8004, respectively. Primers were modified to give EcoRI-, XbaI- or HindIII-compatible ends (underlined) (Additional file 10: Table S5). Two fragments were cloned together into the vector pK18mobsacB, and then the recombinant plasmid was introduced into the *Xcc* strain 8004 by triparental conjugation. The trans-conjugants were screened for selective agar plates containing 5% sucrose. The obtained *pilG* deletion mutant was further confirmed by PCR and named ΔpilG. Other deletion mutants were constructed in the same way as mutant ΔpilG.

For construction of complemented mutant, a DNA fragment containing the encoding region of *pilG* was amplified by PCR using the primers CGF/R, and then the amplified DNA fragment was cloned into the plasmid pLAFR3 (Additional file 6: Table S1) to generate recombinant plasmid. While the recombinant plasmid was introduced into the mutant ΔpilG by triparental conjugation, generating complemented strain CpilG. Other complemented strains were constructed in the same way as the complemented strain CpilG.

### Motility assay

For examination of swimming motility, strains were stabbed into 0.28% agar plate containing 0.03% Bacto peptone and 0.03% yeast extract using toothpick then incubated at 28 °C for 3 days. To analyse swarming motility, strains grown overnight in NYGB medium (OD_600_ = 1.0) were inoculated onto NY plate containing 2% glucose and 0.6% agar then incubated at 28 °C for 3 days.

### Chemotaxis response assay

To analyse chemotaxis response of *Xcc*, we used a simplified capillary assay [41]. The strains were grown in the NYG medium for overnight and diluted to OD_600_ of 1, and then 100 μl diluted culture was sucked into a disposable pipette tip and the chemotaxis capillary containing the chemoattractant was attached to bacterial suspension in disposable pipette tip steadily at 28 °C. Two hours later, the chemoattractant in the chemotaxis capillary was blown out and diluted to 0.0001, and 100 μl diluted culture was plated onto NYGA plate. Bacterial CFUs were counted after incubating at 28 °C for 3 days. H_2_O was used as a control buffer for test capillary. Relative chemotaxis fraction was calculated CFU in test capillary vs CFU in control buffer capillary.

### Plant inoculation assay

The virulence of *Xcc* to Chinese radish (*Raphanus sativus*) was tested by the leaf-clipping or leaf-spraying method. The strains were grown in the NYG medium for overnight and diluted to OD_600_ of 0.001, and then inoculated to Chinese radish by leaf-clipping or leaf-spraying method. Lesion length was measured or diseased leaves rate was counted for 10 days later.

### Growth curve of *Xcc* strains

For in-NY growth curve, the strains were inoculated into NYG medium with the same final density of 0.01, growth of the strains was diluted and plated on NYG plates at intervals of 4 h. The living cells were counted after 3 days of incubation at 28 °C.

Bacterial in planta growth was tested as previously described [42]. The strains were grown in the NYG medium for overnight and diluted to OD_600_ of 0.001, and then inoculated to Chinese radish by leaf-clipping. At intervals of 1 day, four clipped leaves for every group of inoculated plants were collected and homogenized, and then diluted NYG medium to plate on NYG plates. The colonies were counted after 3 days of incubation at 28 °C.

### EPS assays

To estimate EPS production, the strains were inoculated into 100 mL NY liquid medium containing glucose (2% w/v) at 28 °C, 200 rpm for 5 days. EPS was precipitated from the culture supernatant with ethanol and dried at 55 °C and weighed as described [5]. For analysis of EPS production on plates, the strains were grown in the NYG medium for overnight and diluted to OD_600_ of 1, and then 3 μl diluted culture was inoculated onto the NY plates containing 2% glucose and 2.0% agar. The results were observed after 5 days of incubation at 28 °C.

### Global transcriptional analysis

Total RNA was sent to Majorbio-Shanghai with RNA isolated from cultures of *Xcc* strains grown to an OD_600_ of 1.0 in NYGB medium. and tested quantity and quality on a Nanodrop spectrophotometer ND-8000 and Agilent 2100 bioanalyzer respectively. The sample was subjected to RNA sequence in an Illumina HiSeq2000 platform at a company (Majorbio, Shanghai, China).

### Semi-quantitative PCR

RNA was isolated from *Xcc* strains grown to OD_600_ of 1.0 in NYG medium using Omega’s RNA kit according to manufacturer’s protocol. The cDNA was synthesized from total RNA using Superscript III First Strand Synthesis kit (Invitrogen) according to manufacturer’s protocol. Semi-quantitative PCR was performed with different primer sets using cDNA as templates, 16S rRNA of *Xcc* 8004 as an internal control. Finally, PCR products were separated by electrophoresis on a 1.2% agarose gel.

### Co-immunoprecipitation

In first, plasmid for expressing 3 × Flag-tagged fusion protein (3 × Flag::PilG) was constructed, which was fused with the coding region of 3 × Flag-tag at the 5′ end of the *pilG* gene and cloned into pLAFR3, and then the fused plasmid were introduced into ΔpilG by triparental conjugation. The Flag-tagged fusion protein produced by the fused plasmids in ΔpilG, and complemented strain CpilG for not expressing 3 × Flag-tagged fusion protein as a negative control.

The strains were grown in the NYG medium for overnight and collected by centrifugation at 4 °С 4000 rpm for 10 min, and then washed with PBS buffer. Lysed the cells by resuspending them in 1 ml of ice-cold lysis buffer (25 mM Tris-HCl pH 7.4, 100 mM NaCl, 1 mM EDTA, 1% NP-40, 10% glycerine) containing protease inhibitor and incubated the resuspended cells on ice 2 h. While centrifuged 10 min at 4 °C, and transferred the supernatant into fresh tube. For each sample, it was added 50 μl of ANTI-FLAG (agarose conjugated) and incubated in gently shaking at 4°С for 3 h. Finally washed the agarose six times with ice-cold TBS buffer (or lysis buffer) and eluted the protein complexes by 0.25 M glycine (pH 2.5). Ultimately, we used a western blot assay to confirm 3 × Flag-tagged fusion protein and then placed protein complexes to analyse by mass spectrometry facilities.

### Bacterial two-hybrid assay

Protein-protein interaction was detected with the BacterioMatch II two-hybrid system (Stratagene, La Jolla, CA, USA). For example, the full length *pilG*, *pilH*, *fliM* and *fliN* was amplified by PCR using the total DNA of the *Xcc* wild-type strain 8004 as template and corresponding oligonucleotide set as primer (Additional file 10: Table S5), respectively. The *pilG* and *pilH* were cloned into the pTRG (prey), generating plasmids pTRG*pilG* and pTRG*pilH*. The *fliM* and *fliN* were cloned into the pBT (bait), generating plasmids pBT*fliM* and pBT*fliN*. To test protein-protein interaction, different combination of plasmids was co-transformed into the XL1-Blue MRF′ reporter strain while and interaction was analysed by the manufacturer’s instructions. The reporter strain XL1-Blue MRF′ with different plasmid pairs was grown on no selective plates and double-selective indicator plates containing 5 mM 3-AT and 12.5 μg ml^− 1^ streptomycin. Protein-protein interaction activated the expression of *addA* and *HIS3* genes within the reporter gene cassette of the reporter strain, resulting in resistance to streptomycin and 3-AT.

### Pull-down assay

Pull-down assay was performed by The ProFound pull-down biotinylated protein-protein interaction kit (Pierce, Rockford, IL, USA). The bait protein was biotinylated with sulfo-NHS-LC-biotin in PBS at room temperature for 40 min, and then transferred to the centrifugal column and incubated with 20 μl of streptavidin sepharose™ beads for 40 min at 4 °C. Beads bonded to bait protein were washed three times with TBS. The beads and 120 μg prey protein were mixed and incubated 1 h for shaking gently at 4 °C, prey protein was eluted and analysed by SDS-PAGE followed by Coomassie blue staining after washing beads three times with wash buffer.

## Supplementary information

**Additional file 1:Figure S1.** Identification of PilG and PilH in *Xcc*. (A) Sequence alignment of PilG in *Xcc* and other bacteria. The GenBank number of PilG homologue in *Xanthomonas campestris* pv*. campestris* is AAY48253; that *in Pseudomonas aeruginosa* is NP_249099; that in *Lysobacter enzymogenes* is BAV96685; that in *Acinetobacter baumnnii* is ABO13221. (B) Sequence alignment of PilH in *Xcc* and other bacteria. The GenBank number of PilG homologue in *Xanthomonas campestris* pv. *campestris* is AAY48254; that in *Pseudomonas aeruginosa* is NP_249100; that in *Lysobacter enzymogenes* is BAV96686; that in *Acinetobacter baumnnii* is ABO13220. (C) Sequence alignment of PilG, PilH and CheY (ID: AAY49356) in *Xanthomonas campestris* pv*. campestris* with CheY_Ec_ (ID: 190906748) in *E. coli*.

**Additional file 2:Figure S2.** PilH positively regulates EPS production but PilG not, and they have no effect on the growth in NY medium. (A) Analysis of EPS production on plates. The strains were grown and diluted to OD_600_ of 1, and then 3 μl diluted culture was inoculated onto the NY plates containing 2% glucose and 2.0% agar. The results were observed after 5 days of incubation at 28 °C. (B) Production of EPS in *Xcc* strains. Mean weight of EPS extracted from the wild type strain, the *pilG* mutant, the *pilH* mutant and the corresponding complemented strains. (C) Growth curve of *Xcc* strains in NY medium. The strains were inoculated into NYG medium with the same final density of 0.01, growth of the strains was diluted and plated on NYG plates at intervals of 4 h. The living cells were counted after 3 days of incubation at 28 °C. Significance was tested by Student’s t test (* and ** represent significance at *P* < 0.05 and 0.01, respectively).

**Additional file 3:Figure S3.** Confirmation of RNA-Seq gene expression data by semi-quantitative RT-PCR. Note: Part of the differential expressed genes were performed to confirm the results of RNA-Seq by semi quantitative reverse-transcription PCR (semi RT-PCR). The expression levels of gene transcripts were calculated though the absolute value of log_2_ fold change =1(equivalent to a fold change of 2). ↑: up-regulated; ↓: down-regulated.

**Additional file 4:Figure S4.** Bacterial two-hybrid experiment and pull-down assay demonstrated interaction between FliN and PilG or PilH. (A) Bacterial two-hybrid experiment showed that PilG and PilH interacted with FliN protein. The reporter strain XL1-Blue MRF′ with different plasmid pairs was grown on no selective plates and double-selective indicator plates containing 5 mM 3-AT and 12.5 μg ml^− 1^ streptomycin. Protein-protein interactions activate the expression of *addA* and *HIS3* genes within the reporter gene cassette of the reporter strain, resulting in resistance to streptomycin and 3-AT. (B) The pull-down assay demonstrated interaction between FliN and PilG or PilH in vitro. Lanes: 1, crude extract of BL21/pET30a after induction with IPTG; 2, crude extract of BL21/pET30a-PilG after induction with IPTG; 3, affinity-purified His_6_- PilG protein; 4, crude extract of BL21/pET30a- PilH; 5, affinity-purified His_6_- PilH protein; 6, crude extract of M15/pQE30 after induction with IPTG; 7, crude extract of M15/pQE30- FliN after induction with IPTG; 8, affinity-purified His_6_- FliN protein; 9, pull-down of protein His_6_- PilG by FliN; 10, pull-down of protein His_6_- PilH by FliN; 11, pull-down of protein His_6_- HpaR1 by FliN. M, molecular mass marker.

**Additional file 5:Figure S5.** Mutation in gene *pilI* influences swarming and swimming motility, but mutation in *colS* not. (A) Strains were stabbed into ‘swim’ plate (0.03% Bacto peptone, 0.03% yeast extract and 0.28% agar) then incubated at 28 °C for 3 days or inoculated onto ‘swarm’ plate (NY plate containing 2% glucose and 0.6% agar) then incubated at 28 °C for 3 days. (B) The diameter of the colony 8004, ΔpilI, CpilI, ΔcolS and CcolS on swimming and swarming plates. Significance was tested by Student’s t test (* and ** represent significance at P < 0.05 and 0.01, respectively).

**Additional file 6:Table S1**. Bacterial strains and plasmids used in this work

**Additional file 7:Table S2.** The differential expressed genes of the *pilG* mutant strain ∆pilG in the rich medium NYGB.

**Additional file 8:Table S3.** The differential expressed genes of the *pilH* mutant strain ∆pilH in the rich medium NYGB.

**Additional file 9:Table S4.** The overlap of differential expressed genes of the *pilG* mutant strain ∆pilG and the *pilH* mutant strain ∆pilH in the rich medium NYGB.

**Additional file 10:Table S5.** Primers used in this study.

## Data Availability

All data generated or analyzed during this study are included in this published article.

## Abbreviations

CFU: Colony forming unit
EPS: Extracellular polysaccharide
Kan: Kanamycin
Rif: Rifampicin
Tet: Tetracycline
*Xcc*: *Xanthomonas campestris* pv*. campestris*
