# Supplementary material for: PilG and PilH antagonistically control flagellum-dependent and pili-dependent motility in the phytopathogen Xanthomonas campestris pv. campestris
Source: BMC Microbiol. 2020 Feb 18;20:37. doi: 10.1186/s12866-020-1712-3 (PMC7029496; doi:10.1186/s12866-020-1712-3)
Supplement: Supplementary file 1 — Additional file 1:Figure S1. Identification of PilG and PilH in Xcc. (A) Sequence alignment of PilG in Xcc and other bacteria. The GenBank number of PilG homologue in Xanthomonas campestris pv. campestris is AAY48253; that in Pseudomonas aeruginosa is NP_249099; that in Lysobacter enzymogenes is BAV96685; that in Acinetobacter baumnnii is ABO13221. (B) Sequence alignment of PilH in Xcc and other bacteria. The GenBank number of PilG homologue in Xanthomonas campestris pv. campestris is AAY48254; that in Pseudomonas aeruginosa is NP_249100; that in Lysobacter enzymogenes is BAV96686; that in Acinetobacter baumnnii is ABO13220. (C) Sequence alignment of PilG, PilH and CheY (ID: AAY49356) in Xanthomonas campestris pv. campestris with CheYEc (ID: 190906748) in E. coli. [file 12866_2020_1712_MOESM1_ESM.pdf]

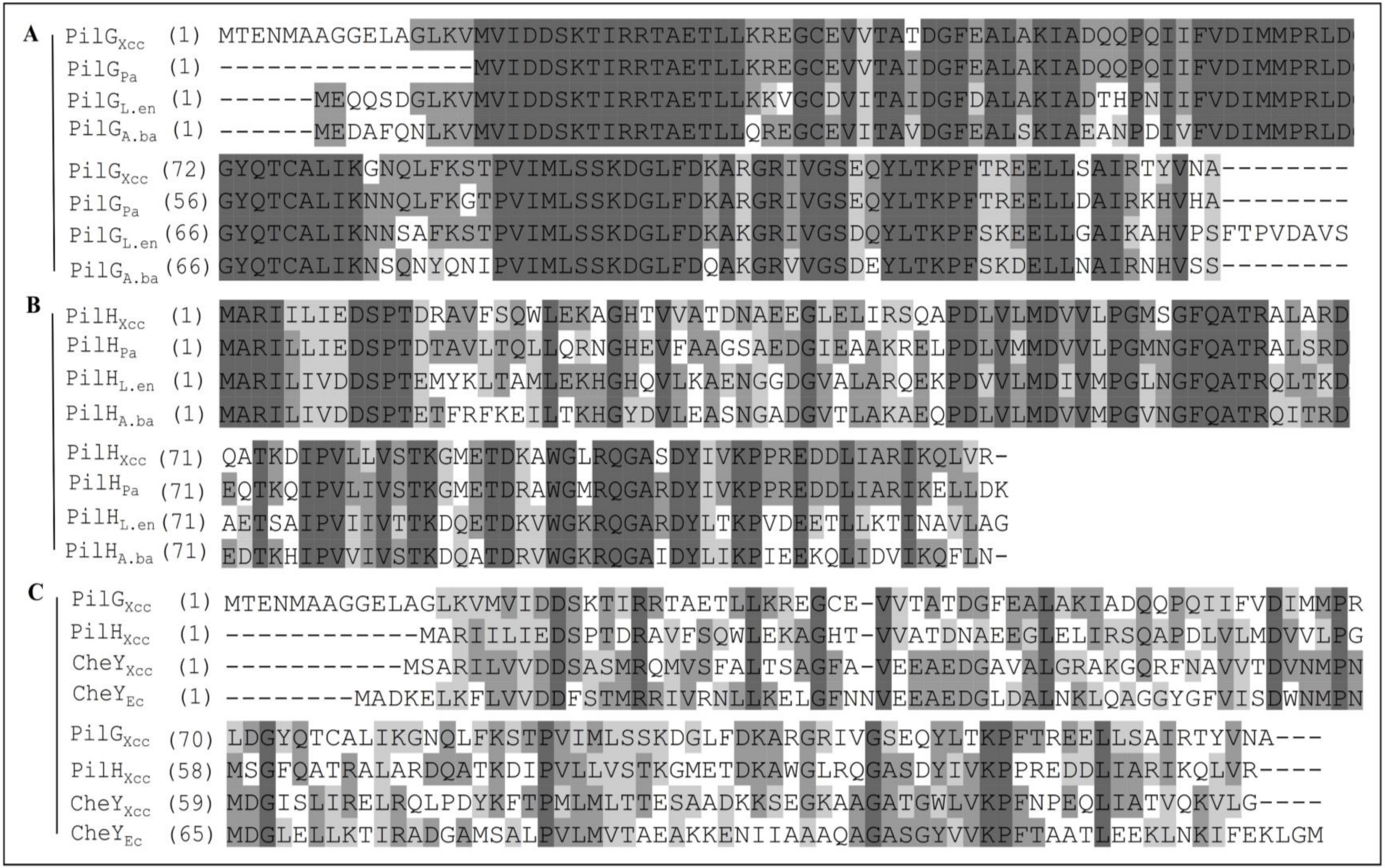
A
PilG$_{Xcc}$ (1) MTENMAAGGELAGLKVMVIDDSKTIRRTAETLLKREGCEVVTATDGFEALAKIADQQPQIIFVDIMMPRLD
PilG$_{Pa}$ (1) ----------------MVIDDSKTIRRTAETLLKREGCEVVTAIDGFEALAKIADQQPQIIFVDIMMPRLD
PilG$_{L.en}$ (1) ------MEQQSDGLKVMVIDDSKTIRRTAETLLKKVGCDVITAIDGFDALAKIADTHPNIIFVDIMMPRLD
PilG$_{A.ba}$ (1) ------MEDAFQNLKVMVIDDSKTIRRTAETLLQREGCEVITAVDGFEALSKIAEANPDIVFVDIMMPRLD
PilG$_{Xcc}$ (72) GYQTCALIKGNQLFKSTPVIMLSSKDGLFDKARGRIVGSEQYLTKPFTREELLSAIRTYVNA--------
PilG$_{Pa}$ (56) GYQTCALIKNNQLFKGTPVIMLSSKDGLFDKARGRIVGSEQYLTKPFTREELLDAIRKHVHA--------
PilG$_{L.en}$ (66) GYQTCALIKNNSAFKSTPVIMLSSKDGLFDKAKGRIVGSDQYLTKPFSKEELLGAIKAHVPSFTPVDAVS
PilG$_{A.ba}$ (66) GYQTCALIKNSQNYQNIPVIMLSSKDGLFDQAKGRVVGSDEYLTKPFSKDELLNAIRNHVSS--------
B
PilH$_{Xcc}$ (1) MARIILIEDSPTDRAVFSQWLEKAGHTVVATDNAEEGLELIRSQAPDLVLMDVVLPGMSGFQATRALARD
PilH$_{Pa}$ (1) MARILLIEDSPTDTAVLTQLLQRNGHEVFAAGSAEDGIEAAKRELPDLVMMDVVLPGMNGFQATRALSRD
PilH$_{L.en}$ (1) MARILIVDDSPTEMYKLTAMLEKHGHQVLKAENGGDGVALARQEKPDVVLMDIVMPGLNGFQATRQLTKD
PilH$_{A.ba}$ (1) MARILIVDDSPTETFRFKEILTKHGYDVLEASNGADGVTLAKAEQPDLVLMDVVMPGVNGFQATRQITRD
PilH$_{Xcc}$ (71) QATKDIPVLLVSTKGMETDKAWGLRQGASDYIVKPPREDDLIARIKQLVR-
PilH$_{Pa}$ (71) EQTKQIPVLIVSTKGMETDRAWGMRQGARDYIVKPPREDDLIARIKELLDK
PilH$_{L.en}$ (71) AETSAIPVIIVTTKDQETDKVWGKRQGARDYLTKPVDEETLLKTINAVLAG
PilH$_{A.ba}$ (71) EDTKHIPVVIVSTKDQATDRVWGKRQGAIDYLIKPIEEKQLIDVIKQFLN-
C
PilG$_{Xcc}$ (1) MTENMAAGGELAGLKVMVIDDSKTIRRTAETLLKREGCE-VVTATDGFEALAKIADQQPQIIFVDIMMPR
PilH$_{Xcc}$ (1) ------------MARIILIEDSPTDRAVFSQWLEKAGHT-VVATDNAEEGLELIRSQAPDLVLMDVVLPG
CheY$_{Xcc}$ (1) -----------MSARILVVDDSASMRQMVSFALTSAGFA-VEEAEDGAVALGRAKGQRFNAVVTDVNMPN
CheY$_{Ec}$ (1) --------MADKELKFLVVDDFSTMRRIVRNLLKELGFNNVEEAEDGLDALNKLQAGGYGFVISDWNMPN
PilG$_{Xcc}$ (70) LDGYQTCALIKGNQLFKSTPVIMLSSKDGLFDKARGRIVGSEQYLTKPFTREELLSAIRTYVNA---
PilH$_{Xcc}$ (58) MSGFQATRALARDQATKDIPVLLVSTKGMETDKAWGLRQGASDYIVKPPREDDLIARIKQLVR----
CheY$_{Xcc}$ (59) MDGISLIRELRQLPDYKFTPMLMLTTESAADKKSEGKAAGATGWLVKPFNPEQLIATVQKVLG----
CheY$_{Ec}$ (65) MDGLELLKTIRADGAMSALPVLMVTAEAKKENIIAAQAGASGYVVKPFTAATLEEKLNKIFEKLGM
