# Supplementary figures and images for: PilG and PilH antagonistically control flagellum-dependent and pili-dependent motility in the phytopathogen Xanthomonas campestris pv. campestris
Source: BMC Microbiol. 2020 Feb 18;20:37. doi: 10.1186/s12866-020-1712-3 (PMC7029496; doi:10.1186/s12866-020-1712-3)

**A**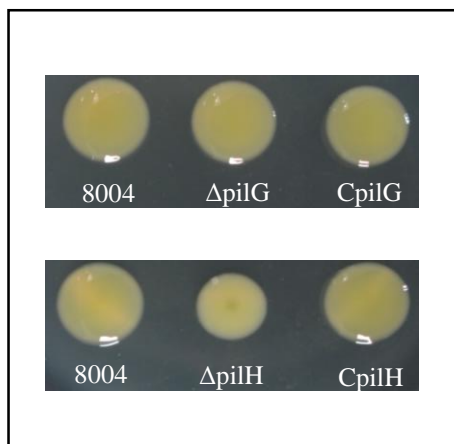**B**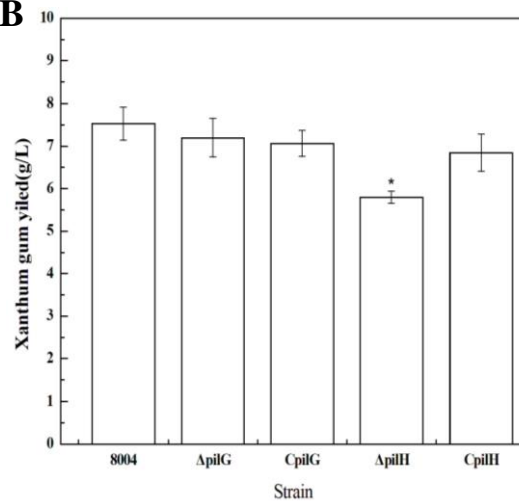**C**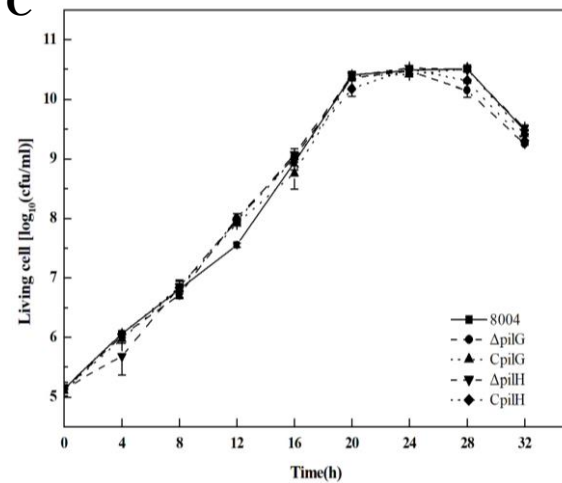

Supplement: Supplementary file 2 — Additional file 2:Figure S2. PilH positively regulates EPS production but PilG not, and they have no effect on the growth in NY medium. (A) Analysis of EPS production on plates. The strains were grown and diluted to OD600 of 1, and then 3 μl diluted culture was inoculated onto the NY plates containing 2% glucose and 2.0% agar. The results were observed after 5 days of incubation at 28 °C. (B) Production of EPS in Xcc strains. Mean weight of EPS extracted from the wild type strain, the pilG mutant, the pilH mutant and the corresponding complemented strains. (C) Growth curve of Xcc strains in NY medium. The strains were inoculated into NYG medium with the same final density of 0.01, growth of the strains was diluted and plated on NYG plates at intervals of 4 h. The living cells were counted after 3 days of incubation at 28 °C. Significance was tested by Student’s t test (* and ** represent significance at P < 0.05 and 0.01, respectively). [file 12866_2020_1712_MOESM2_ESM.pdf]

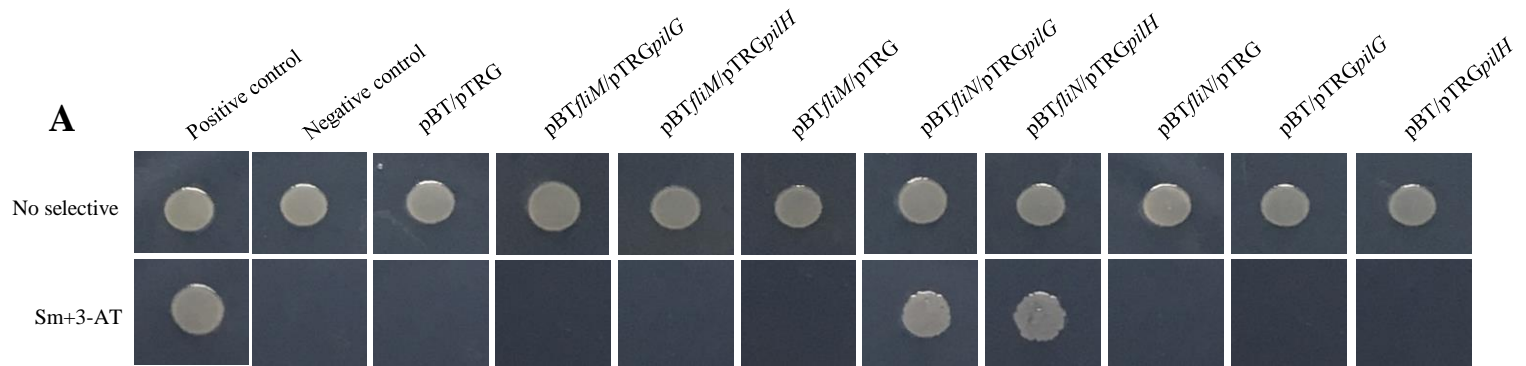

**B**

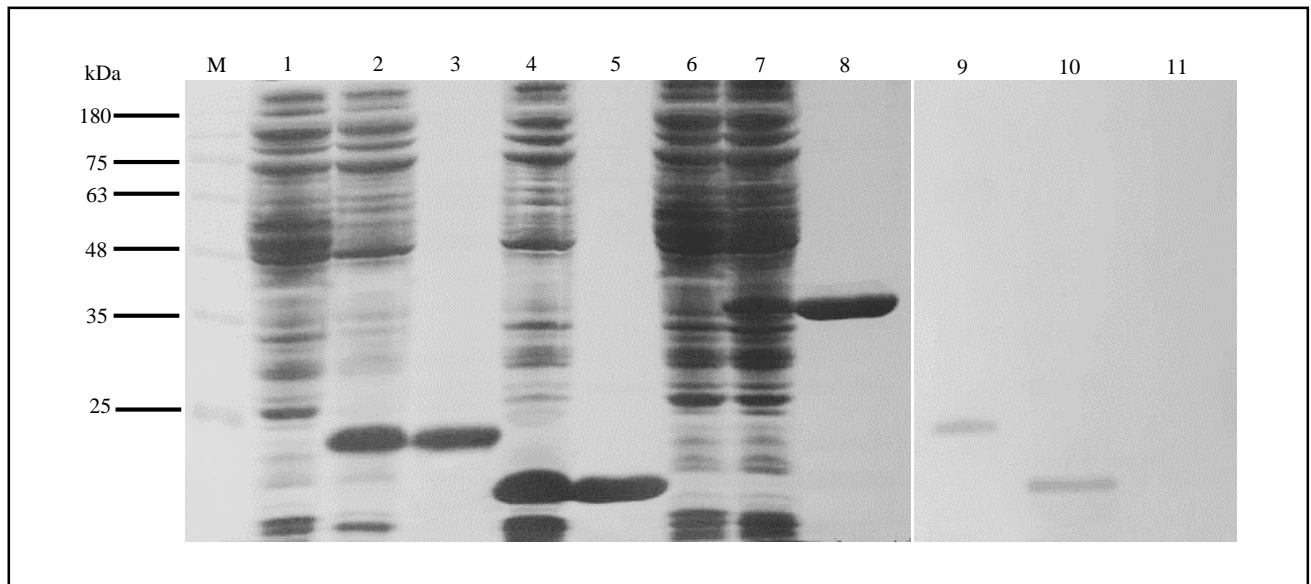

Supplement: Supplementary file 4 — Additional file 4:Figure S4. Bacterial two-hybrid experiment and pull-down assay demonstrated interaction between FliN and PilG or PilH. (A) Bacterial two-hybrid experiment showed that PilG and PilH interacted with FliN protein. The reporter strain XL1-Blue MRF′ with different plasmid pairs was grown on no selective plates and double-selective indicator plates containing 5 mM 3-AT and 12.5 μg ml− 1 streptomycin. Protein-protein interactions activate the expression of addA and HIS3 genes within the reporter gene cassette of the reporter strain, resulting in resistance to streptomycin and 3-AT. (B) The pull-down assay demonstrated interaction between FliN and PilG or PilH in vitro. Lanes: 1, crude extract of BL21/pET30a after induction with IPTG; 2, crude extract of BL21/pET30a-PilG after induction with IPTG; 3, affinity-purified His6- PilG protein; 4, crude extract of BL21/pET30a- PilH; 5, affinity-purified His6- PilH protein; 6, crude extract of M15/pQE30 after induction with IPTG; 7, crude extract of M15/pQE30- FliN after induction with IPTG; 8, affinity-purified His6- FliN protein; 9, pull-down of protein His6- PilG by FliN; 10, pull-down of protein His6- PilH by FliN; 11, pull-down of protein His6- HpaR1 by FliN. M, molecular mass marker. [file 12866_2020_1712_MOESM4_ESM.pdf]

**A**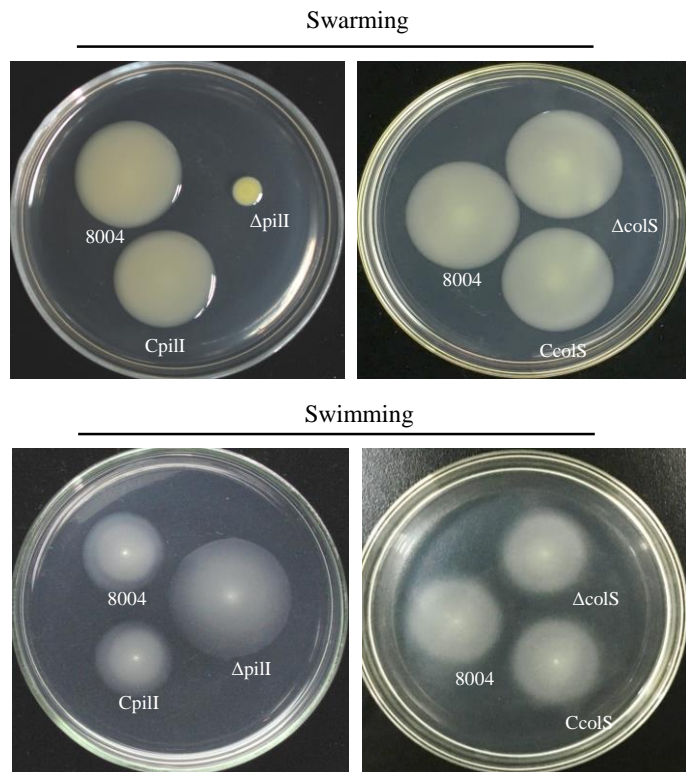**B**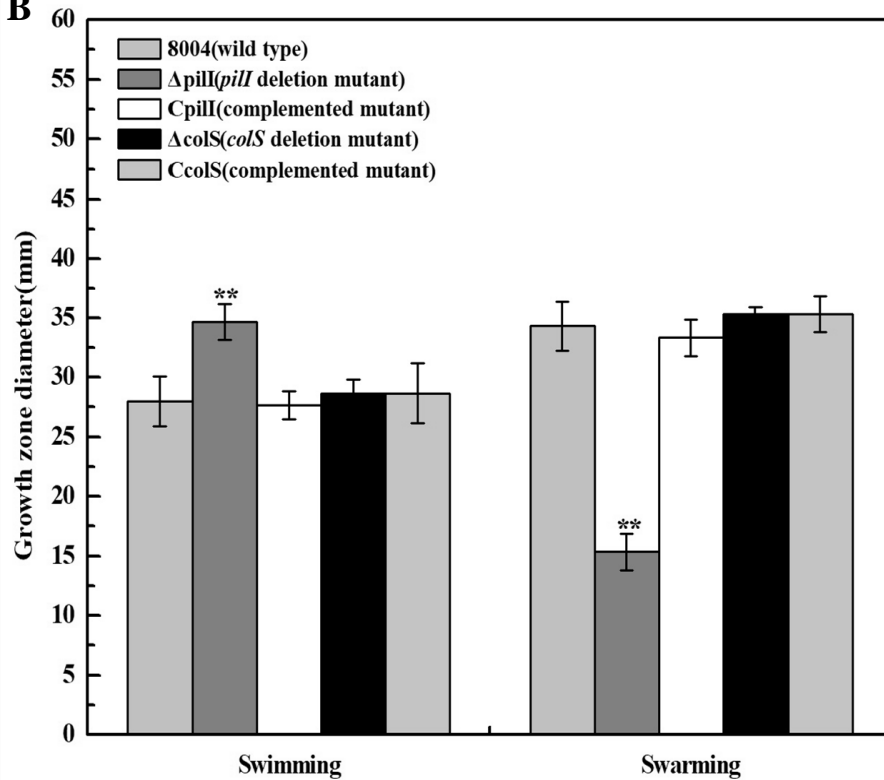

Supplement: Supplementary file 5 — Additional file 5:Figure S5. Mutation in gene pilI influences swarming and swimming motility, but mutation in colS not. (A) Strains were stabbed into ‘swim’ plate (0.03% Bacto peptone, 0.03% yeast extract and 0.28% agar) then incubated at 28 °C for 3 days or inoculated onto ‘swarm’ plate (NY plate containing 2% glucose and 0.6% agar) then incubated at 28 °C for 3 days. (B) The diameter of the colony 8004, ΔpilI, CpilI, ΔcolS and CcolS on swimming and swarming plates. Significance was tested by Student’s t test (* and ** represent significance at P < 0.05 and 0.01, respectively). [file 12866_2020_1712_MOESM5_ESM.pdf]
