# Supplementary material for: PilG and PilH antagonistically control flagellum-dependent and pili-dependent motility in the phytopathogen Xanthomonas campestris pv. campestris
Source: BMC Microbiol. 2020 Feb 18;20:37. doi: 10.1186/s12866-020-1712-3 (PMC7029496; doi:10.1186/s12866-020-1712-3)
Supplement: Supplementary file 3 — Additional file 3:Figure S3. Confirmation of RNA-Seq gene expression data by semi-quantitative RT-PCR. Note: Part of the differential expressed genes were performed to confirm the results of RNA-Seq by semi quantitative reverse-transcription PCR (semi RT-PCR). The expression levels of gene transcripts were calculated though the absolute value of log2 fold change =1(equivalent to a fold change of 2). ↑: up-regulated; ↓: down-regulated. [file 12866_2020_1712_MOESM3_ESM.pdf]

ID	Gene	Description	Expresstion level pilH	pilG	Semi RT-PCR ΔpilH/wt/ΔpilG
XC_2320		methyl-accepting chemotaxis protein I, serine sensor receptor	2.16 ↑	5.10 ↓	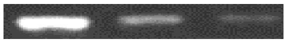
XC_2302	CheY	chemotaxis protein CheY	2.53 ↑	2.20 ↓	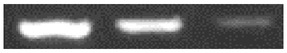
XC_2311		methyl-accepting chemotaxis protein I, serine sensor receptor	2.38 ↑	3.43 ↓	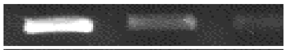
XC_2245		flagellar protein	4.36 ↑	2.83 ↓	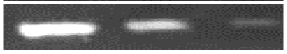
XC_2309		methyl-accepting chemotaxis protein I, serine sensor receptor	2.13 ↑	3.51 ↓	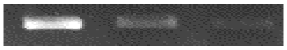
XC_1413		methyl-accepting chemotaxis protein	2.62 ↑	2.39 ↓	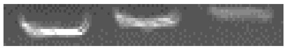
XC_0638		methyl-accepting chemotaxis protein I, serine sensor receptor	2.36 ↑	2.35 ↓	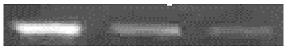
XC_2321	CheR	chemotaxis protein methyltransferase CheR	2.69 ↑	3.71 ↓	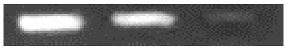
XC_2306		chemotaxis protein	2.45 ↑	3.25 ↓	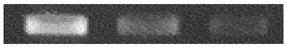
XC_2264	FliJ	flagellar FliJ protein	2.36 ↑	6.73 ↓	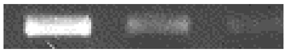
XC_1410	CheR	chemotaxis protein methyltransferase CheR	2.06 ↑	3.23 ↓	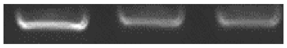
XC_2303	CheA	sensor kinase CheA	2.43 ↑	2.20 ↓	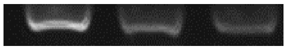
XC_2314		methyl-accepting chemotaxis protein I, serine sensor receptor	4.35 ↑	2.03 ↓	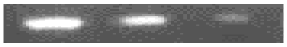
XC_2785		helicase	2.71 ↑	2.23 ↓	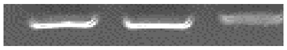
XC_2301		conserved hypothetical protein	2.50 ↑	2.81 ↓	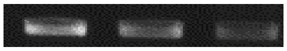
XC_1414	CheA	chemotaxis histidine protein kinase	2.31 ↑	2.10 ↓	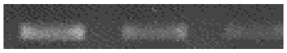
XC_2230		conserved hypothetical protein	2.04 ↑	2.45 ↓	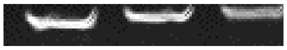
XC_1201	RebB	RebB protein	2.11 ↑	2.89 ↓	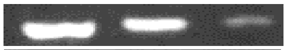
16s					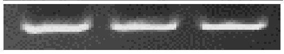
