# Supplementary material for: PilG and PilH antagonistically control flagellum-dependent and pili-dependent motility in the phytopathogen Xanthomonas campestris pv. campestris
Source: BMC Microbiol. 2020 Feb 18;20:37. doi: 10.1186/s12866-020-1712-3 (PMC7029496; doi:10.1186/s12866-020-1712-3)
Supplement: Supplementary file 6 — Additional file 6:Table S1. Bacterial strains and plasmids used in this work [file 12866_2020_1712_MOESM6_ESM.docx]

**Table S1**. Bacterial strains and plasmids used in this work

| Strains or plasmids | Relevant characteristics | Reference or source |
| --- | --- | --- |
| *E. coli* strains |  |  |
| DH5α | Φ80△*lacZM*15 *recA1 endA1 deoR* | Gibco BRL, Life Technologies |
| XL1-Blue MRF' | Reporter Strain, *△(mcrA)183 △(mcrCB-hsdSMR-mrr)173 endA1 hisB supE44* *thi-lrecA gyrA96relAl lac* [F’ *lacIP HIS3 aadA* Kan^r^] | Stratagene |
| XL1-Blue MRF'-pBT*fliM*/pTRG*pilG* | XL1-Blue MRF' harbouring recombinant pBT*fliM* and pTRG*pilG* | This work |
| XL1-Blue MRF' -pBT*fliM*/pTRG*pilH* | XL1-Blue MRF' harbouring recombinant pBT*fliNM* and pTRG*pilH* | This work |
| XL1-Blue MRF'-pBT*fliM*/pTRG | XL1-Blue MRF' harbouring recombinant pBT*fliM* and pTRG | This work |
| XL1-Blue MRF'-pBT/pTRG*pilG* | XL1-Blue MRF' harbouring recombinant pBT and pTRG*pilG* | This work |
| XL1-Blue MRF'-pBT/pTRG*pilH* | XL1-Blue MRF' harbouring recombinant pBT and pTRG*pilH* | This work |
| XL1-Blue MRF'-pBT*fliN*/pTRG | XL1-Blue MRF' harbouring recombinant pBT*fliN* and pTRG | This work |
| XL1-Blue MRF'-pBT/pTRG | XL1-Blue MRF' harbouring recombinant pBT and pTRG | This work |
| XL1-Blue MRF'-pBT*fliN*/pTRG*pilG* | XL1-Blue MRF' harbouring recombinant pBT*fliN* and pTRG*pilG* | This work |
| XL1-Blue MRF'-pBT*fliN*/pTRG*pilH* | XL1-Blue MRF' harbouring recombinant pBT*fliN* and pTRG*pilH* | This work |
| XL1-Blue MRF'-pBT*pilG*/pTRG*3619* | XL1-Blue MRF' harbouring recombinant pBT*pilG* and pTRG*3619* | This work |
| XL1-Blue MRF' - pBT*pilG* /pTRG*pilN* | XL1-Blue MRF' harbouring recombinant pBT*pilG* and pTRG*pilN* | This work |
| XL1-Blue MRF'- pBT*pilG* /pTRG*cheA* | XL1-Blue MRF' harbouring recombinant pBT*pilG* and pTRG*cheA* | This work |
| XL1-Blue MRF'- pBT*pilG* /pTRG*pilU* | XL1-Blue MRF' harbouring recombinant pBT*pilG* and pTRG*pilU* | This work |
| XL1-Blue MRF'- pBT*pilG* /pTRG*pilR* | XL1-Blue MRF' harbouring recombinant pBT*pilG* and pTRG*pilR* | This work |
| XL1-Blue MRF'- pBT*pilG* /pTRG*1163* | XL1-Blue MRF' harbouring recombinant pBT*pilG* and pTRG*1163* | This work |
| XL1-Blue MRF'- pBT*pilG* /pTRG | XL1-Blue MRF' harbouring recombinant pBT*pilG* and pTRG | This work |
| XL1-Blue MRF'-pBT/pTRG*3619* | XL1-Blue MRF' harbouring recombinant pBT and pTRG*3619* | This work |
| XL1-Blue MRF' - pBT /pTRG*pilN* | XL1-Blue MRF' harbouring recombinant pBT and pTRG*pilN* | This work |
| XL1-Blue MRF'- pBT /pTRG*cheA* | XL1-Blue MRF' harbouring recombinant pBT and pTRG*cheA* | This work |
| XL1-Blue MRF'- pBT /pTRG*pilU* | XL1-Blue MRF' harbouring recombinant pBT and pTRG*pilU* | This work |
| XL1-Blue MRF'- pBT /pTRG*pilR* | XL1-Blue MRF' harbouring recombinant pBT and pTRG*pilR* | This work |
| XL1-Blue MRF'- pBT /pTRG*1163* | XL1-Blue MRF' harbouring recombinant pBT and pTRG*1163* | This work |
| XL1-Blue MRF'-pBT*pilH*/pTRG*1355* | XL1-Blue MRF' harbouring recombinant pBT*pilH* and pTRG*1355* | This work |
| XL1-Blue MRF' - pBT*pilH* /pTRG*colS* | XL1-Blue MRF' harbouring recombinant pBT*pilH* and pTRG*colS* | This work |
| XL1-Blue MRF'- pBT*pilH* /pTRG*pilI* | XL1-Blue MRF' harbouring recombinant pBT*pilH* and pTRG*pilI* | This work |
| XL1-Blue MRF'- pBT*pilH* /pTRG*1378* | XL1-Blue MRF' harbouring recombinant pBT*pilH* and pTRG*1378* | This work |
| XL1-Blue MRF'- pBT*pilH* /pTRG*3262* | XL1-Blue MRF' harbouring recombinant pBT*pilH* and pTRG*3262* | This work |
| XL1-Blue MRF'- pBT*pilH* /pTRG*phoP* | XL1-Blue MRF' harbouring recombinant pBT*pilH* and pTRG*phoP* | This work |
| XL1-Blue MRF'- pBT*pilH* /pTRG*3597* | XL1-Blue MRF' harbouring recombinant pBT*pilH* and pTRG*3597* | This work |
| XL1-Blue MRF'- pBT*pilH* /pTRG | XL1-Blue MRF' harbouring recombinant pBT*pilH* and pTRG | This work |
| XL1-Blue MRF'-pBT/pTRG*1355* | XL1-Blue MRF' harbouring recombinant pBT and pTRG*1355* | This work |
| XL1-Blue MRF' - pBT /pTRG*colS* | XL1-Blue MRF' harbouring recombinant pBT and pTRG*colS* | This work |
| XL1-Blue MRF'- pBT /pTRG*pilI* | XL1-Blue MRF' harbouring recombinant pBT and pTRG*pilI* | This work |
| XL1-Blue MRF'- pBT /pTRG*1378* | XL1-Blue MRF' harbouring recombinant pBT and pTRG*1378* | This work |
| XL1-Blue MRF'- pBT /pTRG*3262* | XL1-Blue MRF' harbouring recombinant pBT and pTRG*3262* | This work |
| XL1-Blue MRF'- pBT /pTRG*phoP* | XL1-Blue MRF' harbouring recombinant pBT and pTRG*phoP* | This work |
| XL1-Blue MRF'- pBT /pTRG*3597* | XL1-Blue MRF' harbouring recombinant pBT and pTRG*3597* | This work |
| BL21(DE3) | F^-^ *ompT gal dcm lon hsdS_B_* (*r^-^_B_ m^-^_B_*) λ(DE3) | Novagen, Germany |
| M15 | *lac ara gal mtl recA1 uvr1* [pREP4 *lacI* Kan^r^] | Qiagen, Germany |
| BL21/pET30a-PilG | BL21(DE3) harbouring recombinant pET30a-PilG | This work |
| BL21/pET30a-PilH | BL21(DE3) harbouring recombinant pET30a-PilH | This work |
| M15/pQE30-FliN | M15 harbouring recombinant pQE30-FliN | This work |
| *Xanthomonas campestris* pv. *campestris* strains |  |  |
| 8004 | Wild-type strain, Rif^r^ | Daniels *et al.*, 1984 |
| ΔpilG | As 8004, but *pilG* gene (*XC_1183*) deleted, non-polar effect. Rif^r^ | This work |
| C∆pilG | ∆pilG harboring the recombinant plasmid pLC*pilG*. Rif^r^ Tet^r^ | This work |
| ΔpilH | As 8004, but *pilH* gene (*XC_1184*) deleted, non-polar effect. Rif^r^ | This work |
| C∆pilH | ∆pilH harboring the recombinant plasmid pLC*pilH*. Rif^r^ Tet^r^ | This work |
| ΔfliM | As 8004, but *fliM* gene (*XC_2267*) deleted, non-polar effect. Rif^r^ | This work |
| C∆fliM | ∆fliM harboring the recombinant plasmid pLC*fliM*. Rif^r^ Tet^r^ | This work |
| ΔfliN | As 8004, but *fliN* gene (*XC_2268*) deleted, non-polar effect. Rif^r^ | This work |
| C∆fliN | ∆fliN harboring the recombinant plasmid pLC*fliN*. Rif^r^ Tet^r^ | This work |
| ΔpilB | As 8004, but *pilB* gene (*XC_1060*) deleted, non-polar effect. Rif^r^ | This work |
| C∆pilB | ∆pilB harboring the recombinant plasmid pLC*pilB*. Rif^r^ Tet^r^ | This work |
| ΔcheA | As 8004, but *cheA* gene (*XC_1414*) deleted, non-polar effect. Rif^r^ | This work |
| C∆cheA | ∆cheA harboring the recombinant plasmid pLC*cheA*. Rif^r^ Tet^r^ | This work |
| Δ2306 | As 8004, but *pilG* gene (*XC_1183*) deleted, non-polar effect. Rif^r^ | This work |
| C∆2306 | ∆pilG harboring the recombinant plasmid pLC*pilG*. Rif^r^ Tet^r^ | This work |
| ΔcheY | As 8004, but *cheY* gene (*XC_2302*) deleted, non-polar effect. Rif^r^ | This work |
| C∆cheY | ∆cheY harboring the recombinant plasmid pLC*cheY*. Rif^r^ Tet^r^ | This work |
| ΔpilI | As 8004, but *pilI* gene (*XC_1185*) deleted, non-polar effect. Rif^r^ | This work |
| C∆pilI | ∆pilI harboring the recombinant plasmid pLC*pilI*. Rif^r^ Tet^r^ | This work |
| ΔcolS | As 8004, but *colS* gene (*XC_1050*) deleted, non-polar effect. Rif^r^ | This work |
| CΔcolS | ∆pilI harboring the recombinant plasmid pLC*colS*. Rif^r^ Tet^r^ | This work |
| ΔrsmA | As 8004, but rsmA gene (*XC_2506*) deleted, non-polar effect. Rif^r^ | Chao *et al*.,2008 |
| ∆pilG/pPilG-Flag | ∆pilG harboring the recombinant plasmid pPilG-Flag. Rif^r^ Tet^r^ | This work |
| ∆pilG/pPilH-Flag | ∆pilH harboring the recombinant plasmid pPilH-Flag. Rif^r^ Tet^r^ | This work |
| Plasmids |  |  |
| pLAFR3 | Broad host range cloning vector. Tet^r^ | Staskawicz *et al*.,1987 |
| pLC*pilG* | pLAFR3 containing an DNA fragment of the *pilG* gene (*XC_1183*) of *Xcc* strain. Tet^r^ | This work |
| pLC*pilH* | pLAFR3 containing an DNA fragment of the *pilH* gene (*XC_1184*) of *Xcc* strain. Tet^r^ | This work |
| pLC*fliM* | pLAFR3 containing an DNA fragment of the *fliM* gene (*XC_2267*) of *Xcc* strain. Tet^r^ | This work |
| pLC*fliN* | pLAFR3 containing an DNA fragment of the *fliN* gene (*XC_2268*) of *Xcc* strain. Tet^r^ | This work |
| pLC*pilB* | pLAFR3 containing an DNA fragment of the *pilB* gene (*XC_1060*) of *Xcc* strain. Tet^r^ | This work |
| pLC*cheA* | pLAFR3 containing an DNA fragment of the *cheA* gene (*XC_1414*) of *Xcc* strain. Tet^r^ | This work |
| pLC*2306* | pLAFR3 containing an DNA fragment of the gene *XC_2306* of *Xcc* strain. Tet^r^ | This work |
| pLC*cheY* | pLAFR3 containing an DNA fragment of the *cheY* gene (*XC_2302*) of *Xcc* strain. Tet^r^ | This work |
| pLC*pilI* | pLAFR3 containing an DNA fragment of the *pilI* gene (*XC_1185*) of *Xcc* strain. Tet^r^ | This work |
| pLC*colS* | pLAFR3 containing an DNA fragment of the *colS* gene (*XC_1050*) of *Xcc* strain. Tet^r^ | This work |
| pRK2073 | Helper plasmid, Tra^+^, Mob^+^, ColE1, Spc^r^. | Leong *et al*.,1982 |
| pK18*mobsacB* | pUC18 derivative, *lacZα*, *sacB*, Kan^r^, *mob* site. Allelic exchange vector (Suicidal vector carrying *sacB* gene for mutagenesis). | Schäfer *et al*., 1994 |
| pKSΔ*pilG* | pK18*mobsacB* containing fragments flanking *pilG* gene. Kan^r^ | This work |
| pKSΔ*pilH* | pK18*mobsacB* containing fragments flanking *pilH* gene. Kan^r^ | This work |
| pKSΔ*fliM* | pK18*mobsacB* containing fragments flanking *fliM* gene. Kan^r^ | This work |
| pKSΔ*fliN* | pK18*mobsacB* containing fragments flanking *fliN* gene. Kan^r^ | This work |
| pKSΔ*pilB* | pK18*mobsacB* containing fragments flanking *pilB* gene. Kan^r^ | This work |
| pKSΔ*cheA* | pK18*mobsacB* containing fragments flanking *cheA* gene. Kan^r^ | This work |
| pKSΔ*2306* | pK18*mobsacB* containing fragments flanking *XC_2306* gene. Kan^r^ | This work |
| pKSΔ*cheY* | pK18*mobsacB* containing fragments flanking *cheY* gene. Kan^r^ | This work |
| pBT | *cat* gene, p15A origin of replication and λ cI ORF. Cat^r^ | Stratagene |
| pTRG | the *tet* gene, ColE1 origin of replication, and RNA polymerase α subunit ORF | Stratagene |
| pBT*fliM* | containing fragments flanking *fliM* gene. Cat^r^ | This work |
| pBT*fliN* | containing fragments flanking *fliN* gene. Cat^r^ | This work |
| pTRG*pliG* | containing fragments flanking *pilG* gene. Tc^r^ | This work |
| pTRG*pilH* | containing fragments flanking *pilH* gene. Tc^r^ | This work |
| pBT*pilG* | containing fragments flanking *pilG* gene. Cat^r^ | This work |
| pBT*pilH* | containing fragments flanking *pilH* gene. Cat^r^ | This work |
| pTRG*3619* | containing fragments flanking *XC_3619* gene. Tc^r^ | This work |
| pTRG*pilN* | containing fragments flanking *pilN* gene. Tc^r^ | This work |
| pTRG*cheA* | containing fragments flanking *XC_2284* gene. Tc^r^ | This work |
| pTRG*pilU* | containing fragments flanking *pilU* gene. Tc^r^ | This work |
| pTRG*pilR* | containing fragments flanking *pilR* gene. Tc^r^ | This work |
| pTRG*1163* | containing fragments flanking *XC_1163* gene. Tc^r^ | This work |
| pTRG*1355* | containing fragments flanking *XC_1355* gene. Tc^r^ | This work |
| pTRG*colS* | containing fragments flanking *colS* gene. Tc^r^ | This work |
| pTRG*pilI* | containing fragments flanking *pilI* gene. Tc^r^ | This work |
| pTRG*1378* | containing fragments flanking *XC_1378* gene. Tc^r^ | This work |
| pTRG*3262* | containing fragments flanking *XC_3262* gene. Tc^r^ | This work |
| pTRG*phoP* | containing fragments flanking *phoP* gene. Tc^r^ | This work |
| pTRG*3597* | containing fragments flanking *XC_3597* gene. Tc^r^ | This work |
| pET-30a | Expression vector, allow the production of fusion proteins containing amino terminal 6×His-tagged sequences. Kanr | Novagen, Germany |
| pQE-30 Xa | Expression vector, allow the production of fusion proteins containing amino terminal 6xHis-tagged sequences. Amp^r^ | Qiagen, Germany |
| pET30a-PilG | pET-30a containing an 405-bp fragment of *pilG* gene. | This work |
| pET30a-PilH | pET-30a containing an 363-bp fragment of *pilH* gene. | This work |
| pQE30-FliN | pET-30a containing an 381-bp fragment of *fliN* gene. | This work |

^a^Rif^r^, Kan^r^, Tet^r^, Cat^r^, Amp^r^, and Spc^r^ indicate resistance to rifampicin, kanamycin, tetracycline, spectinomycin, ampicillin, and chloramphenicol, respectively.

**References**

Daniels, M.J., Barber, C.E., Turner, P.C., Sawczyc, M.K., Byrde, R.J.W., and Fielding, A.H. (1984) Cloning of genes involved in pathogenicity of *Xanthomonas campestris* pv. *campestris* using the broad host range cosmid pLAFR1. *EMBO J* **3:** 3323–3328.

Leong, S.A., Ditta, G.S., and Helinski, D.R. (1982) Heme biosynthesis in *Rhizobium*. Identification of a cloned gene coding for delta-aminolevulinic acid synthetase from *Rhizobium meliloti*. *J Biol Chem* **257:** 8724–8730.

Chao NX, Wei K, Chen Q, Meng QL, Tang DJ, He YQ, Lu GT, Jiang BL, Liang XX, Feng JX, Chen BS, and Tang JL. (2008) The *rsmA*-like gene *rsmA_Xcc_* of *Xanthomonas campestris* pv. *campestris* is involved in the control of various cellular processes, including pathogenesis. *MPMI* **21:**411-423.

Schäfer, A., Tauch, A., Jäger, W., Kalinowski, J., Thierbach, G., and Pühler, A. (1994) Small mobilizable multi-purpose cloning vectors derived from the *Escherichia coli* plasmids pK18 and pK19: selection of defined deletions in the chromosome of *Corynebacterium glutamicum*. *Gene* **145:** 69–73.

Staskawicz, B., Dahlbeck, D., Keen, N., and Napoli, C. (1987) Molecular characterization of cloned avirulence genes fromrace 0 and race 1 of *Pseudomonas syringae* pv. *glycinea*. *J Bacteriol* **169:** 5789–5794.
