# Supplementary material for: PilG and PilH antagonistically control flagellum-dependent and pili-dependent motility in the phytopathogen Xanthomonas campestris pv. campestris
Source: BMC Microbiol. 2020 Feb 18;20:37. doi: 10.1186/s12866-020-1712-3 (PMC7029496; doi:10.1186/s12866-020-1712-3)
Supplement: Supplementary file 7 — Additional file 7:Table S2. The differential expressed genes of the pilG mutant strain ∆pilG in the rich medium NYGB. [file 12866_2020_1712_MOESM7_ESM.docx]

**Table S2.** The differential expressed genes of the *pilG* mutant strain ∆pilG in the rich medium NYGB.

| Function Category | Gene ID | Name | Annotation | fold change |
| --- | --- | --- | --- | --- |
| Biosynthesis of cofactors, prosthetic groups, carriers | *XC_0294* | *allC* | N-carbamyl-L-amino acid amidohydrolase | -1.05 |
|  | *XC_2957* | *hprT* | hypoxanthine phosphoribosyltransferase | -1.06 |
|  | *XC_2299* | *parA* | chromosome partitioning protein | -1.61 |
|  | *XC_3808* | *lpxL* | Kdo2-lipid IVA lauroyltransferase | -1.05 |
|  | *XC_1607* | *truB* | tRNA pseudouridine55 synthase | -1.19 |
|  | *XC_3710* | *rsmC* | 16S rRNA (guanine1207-N2)-methyltransferase | 1.06 |
| Cellular processes | *XC_2298* | *motB* | chemotaxis protein MotB | -1.94 |
|  | *XC_2265* | *fliK* | flagellar proteinFliK | -2.88 |
|  | *XC_2264* | *fliJ* | flagellar FliJ protein | -2.75 |
|  | *XC_2245* | *fliC* | flagellar protein | -1.5 |
|  | *XC_2263* | *fliI* | flagellum-specific ATP synthase | -2.16 |
|  | *XC_2231* | *flgM* | flagellar protein | -1.25 |
|  | *XC_2246* | *fliD* | flagellar protein | -1.03 |
|  | *XC_2247* | *fliS* | flagellar protein FliS | -1.07 |
| Cell envelope and cell structure | *XC_2098* |  | ribonuclease E | -2.37 |
| Fatty acid and phospholipid meatbolism | *XC_0396* |  | oxidoreductase | -1.09 |
| Energy and carbon metabolism | *XC_0839* | *ilvM* | acetolactate synthase II small subunit | -1.9 |
|  | *XC_2487* | *dgoA* | 2-dehydro-3-deoxyphosphogalactonate aldolase | -1.62 |
|  | *XC_2177* | *nirB* | nitrite reductase (NADH) large subunit | 1.82 |
| Mobile genetic elements | *XC_2007* |  | transposase | 2.32 |
|  | *XC_0412* |  | putative transposase | 2.43 |
|  | *XC_3804* |  | ISxac3 transposase | 2.64 |
| Regulatory functions | *XC_0257* |  | transcriptional regulator acrR family | -1.46 |
|  | *XC_1102* |  | AraC family transcriptional regulator | -1.11 |
|  | *XC_0750* |  | GntR family transcriptional regulator | -5.96 |
|  | *XC_0072* |  | transcriptional regulator | 4.68 |
|  | *XC_2157* | *nemR* | TetR/AcrR family transcriptional regulator | 4.56 |
|  | *XC_4306* |  | transcriptional regulator | -1.21 |
|  | *XC_4254* | *slyA* | MarR family transcriptional regulator | -1.48 |
| Signal transduction | *XC_2320* | *mtsr* | methyl-accepting chemotaxis protein I, serine sensor receptor | -2.35 |
|  | *XC_2318* | *cheW* | purine-binding chemotaxis protein CheW | -1.94 |
|  | *XC_2311* | *tsr* | ethyl-accepting chemotaxis protein I, serine sensor receptor | -1.78 |
|  | *XC_2309* | *tsr* | methyl-accepting chemotaxis protein I, serine sensor receptor | -1.81 |
|  | *XC_2321* | *cheR* | chemotaxis protein methyltransferase CheR | -1.89 |
|  | *XC_1410* | *cheR* | chemotaxis protein methyltransferase CheR | -1.69 |
|  | *XC_0638* | *tsr* | methyl-accepting chemotaxis protein I, serine sensor receptor | -1.23 |
|  | *XC_2303* | *cheA* | chemotaxis family, sensor kinase CheA | -1.14 |
|  | *XC_1412* | *cheW* | chemotaxis protein CheW | -1.56 |
|  | *XC_2306* |  | chemotaxis protein | -1.7 |
|  | *XC_2223* | *mcp* | methyl-accepting chemotaxis protein | -1.1 |
|  | *XC_0286* | *tsr* | methyl-accepting chemotaxis protein I, serine sensor receptor | -1.3 |
|  | *XC_2302* | *cheY* | chemotaxis family, chemotaxis protein CheY | -1.14 |
|  | *XC_1413* | *mcp* | methyl-accepting chemotaxis protein | -1.26 |
|  | *XC_2313* | *tsr* | methyl-accepting chemotaxis protein I, serine sensor receptor | -1.28 |
|  | *XC_0637* |  | histidine kinase/response regulator hybrid protein | -1.35 |
|  | *XC_2314* | *tsr* | methyl-accepting chemotaxis protein I, serine sensor receptor | -1.02 |
|  | *XC_2315* | *mcp* | methyl-accepting chemotaxis protein | -1.35 |
|  | *XC_2323* | *cheB* | chemotaxis family, protein-glutamate methylesterase | -1.05 |
|  | *XC_1414* | *cheA* | chemotaxis family, sensor kinase CheA | -1.07 |
|  | *XC_1411* |  | response regulator | -2.3 |
| Translation | *XC_3339* | *rplD* | 50S ribosomal protein L4 | -1.04 |
|  | *XC_3337* | *rplB* | 50S ribosomal protein L2 | -1.09 |
|  | *XC_3350* | *rplA* | 50S ribosomal protein L1 | -1.01 |
|  | *XC_2863* | *rpsB* | 30S ribosomal protein S2 | -1.13 |
| Transport | *XC_1341* |  | TonB-dependent receptor | -1.06 |
|  | *XC_0744* |  | type II secretion system protein I | 1.2 |
|  | *XC_3570* |  | general secretion pathway protein H | -1 |
|  | *XC_3788* |  | ABC transporter ATP-binding protein | -1.19 |
| Undefined category | *XC_1201* |  | RebB protein | -1.53 |
|  | *XC_2800* |  | aklaviketone reductase | -2.3 |
|  | *XC_2109* |  | minor coat protein | 9.14 |
|  | *XC_2785* |  | helicase | -1.16 |
|  | *XC_0471* |  | N-acetyltransferase | -1.08 |
|  | *XC_0577* | *mdcB* | CitG protein | -1.57 |
|  | *XC_0516* |  | CDP-diacylglycerol-glycerol-3-phosphate 3-phosphatidyltransferase-related protein | -1.37 |
|  | *XC_0574* | *mdcD* | malonate decarboxylase beta subunit | -1.16 |
|  | *XC_0434* |  | component of multidrug efflux system | -1.42 |
|  | *XC_2551* |  | hexosyltransferase | -1 |
|  | *XC_4305* |  | oxidoreductase | -1.24 |
| hypothetical protein | *XC_2319* |  | conserved hypothetical protein | -1.82 |
|  | *XC_1415* |  | conserved hypothetical protein | -2.82 |
|  | *XC_0262* |  | conserved hypothetical protein | -3.08 |
|  | *XC_2414* |  | hypothetical protein | -1.51 |
|  | *XC_2416* |  | hypothetical protein | -1.75 |
|  | *XC_2415* |  | conserved hypothetical protein | -1.84 |
|  | *XC_2036* |  | conserved hypothetical protein | 3.07 |
|  | *XC_2050* |  | conserved hypothetical protein | 2.48 |
|  | *XC_2305* |  | conserved hypothetical protein | -2.53 |
|  | *XC_3525* |  | conserved hypothetical protein | -1.6 |
|  | *XC_0523* |  | conserved hypothetical protein | -1.96 |
|  | *XC_2412* |  | hypothetical protein | -1.14 |
|  | *XC_2301* |  | conserved hypothetical protein | -1.49 |
|  | *XC_4034* |  | conserved hypothetical protein | -1.26 |
|  | *XC_3178* |  | conserved hypothetical protein | -1.4 |
|  | *XC_0661* |  | conserved hypothetical protein | -1.89 |
|  | *XC_3668* |  | conserved hypothetical protein | -1.91 |
|  | *XC_2411* |  | conserved hypothetical protein | -1.02 |
|  | *XC_0362* |  | conserved hypothetical protein | -1.26 |
|  | *XC_0078* |  | conserved hypothetical protein | 1.25 |
|  | *XC_2230* |  | conserved hypothetical protein | -1.29 |
|  | *XC_2233* |  | conserved hypothetical protein | -1.16 |
|  | *XC_0220* |  | conserved hypothetical protein | -2.12 |
|  | *XC_3367* |  | conserved hypothetical protein | -1.3 |
|  | *XC_1353* |  | conserved hypothetical protein | -1.44 |
|  | *XC_2365* |  | conserved hypothetical protein | -1.27 |
|  | *XC_2861* |  | conserved hypothetical protein | -1.7 |
|  | *XC_0852* |  | conserved hypothetical protein | 1.09 |
|  | *XC_2830* |  | conserved hypothetical protein | -1.23 |
|  | *XC_2788* |  | conserved hypothetical protein | -1.06 |
|  | *XC_3784* |  | conserved hypothetical protein | -1.67 |
|  | *XC_3893* |  | conserved hypothetical protein | -1.81 |
|  | *XC_2786* |  | conserved hypothetical protein | -1.11 |
|  | *XC_0090* |  | conserved hypothetical protein | -1.79 |
|  | *XC_1453* |  | conserved hypothetical protein | 1.1 |
|  | *XC_2249* |  | conserved hypothetical protein | -1.08 |
|  | *XC_4143* |  | conserved hypothetical protein | -1.64 |
|  | *XC_3128* |  | conserved hypothetical protein | -1.01 |
|  | *XC_4246* |  | conserved hypothetical protein | -1.66 |
|  | *XC_2550* |  | conserved hypothetical protein | 1.37 |
|  | *XC_0233* |  | conserved hypothetical protein | -1.2 |
|  | *XC_3108* |  | conserved hypothetical protein | -1.05 |
|  | *XC_0788* |  | conserved hypothetical protein | -1.17 |
|  | *XC_1984* |  | hypothetical protein | -1.41 |
|  | *XC_1340* |  | conserved hypothetical protein | -1.22 |
|  | *XC_3961* |  | conserved hypothetical protein | 1.19 |
|  | *XC_1685* |  | hypothetical protein | 6.41 |
|  | *XC_3000* |  | conserved hypothetical protein | 7.35 |
|  | *XC_2057* |  | conserved hypothetical protein | 1.18 |
|  | *XC_1458* |  | hypothetical protein | -6.91 |
|  | *XC_3462* |  | conserved hypothetical protein | 1.11 |
|  | *XC_3755* |  | conserved hypothetical protein | -1.1 |
|  | *XC_1337* |  | conserved hypothetical protein | -1.11 |
|  | *XC_0902* |  | conserved hypothetical protein | -1.18 |
|  | *XC_1763* |  | conserved hypothetical protein | -1.71 |
|  | *XC_2034* |  | conserved hypothetical protein | 1.5 |
|  | *XC_2595* |  | conserved hypothetical protein | 1.23 |
|  | *XC_3783* |  | conserved hypothetical protein | -1.15 |
|  | *XC_1460* |  | conserved hypothetical protein | -1.1 |
|  | *XC_3049* |  | conserved hypothetical protein | -1.02 |
|  | *XC_4300* |  | conserved hypothetical protein | -1.18 |
|  | *XC_2353* |  | conserved hypothetical protein | -1.87 |
|  | *XC_3297* |  | conserved hypothetical protein | -1.43 |
|  | *XC_1202* |  | conserved hypothetical protein | -1.04 |
|  | *XC_1281* |  | conserved hypothetical protein | -1.28 |
|  | *XC_0560* |  | conserved hypothetical protein | 1.02 |
|  | *XC_2224* |  | conserved hypothetical protein | -1.42 |
|  | *XC_3549* |  | conserved hypothetical protein | -1.13 |
|  | *XC_2025* |  | conserved hypothetical protein | 1.32 |
|  | *XC_2058* |  | conserved hypothetical protein | -1.55 |
|  | *XC_4111* |  | conserved hypothetical protein | -1.03 |
|  | *XC_4116* |  | conserved hypothetical protein | -1 |
|  | *XC_1194* |  | conserved hypothetical protein | -1.05 |
|  | *XC_4285* |  | conserved hypothetical protein | -1.34 |
|  | *XC_2535* |  | conserved hypothetical protein | -1.22 |
|  | *XC_3940* |  | conserved hypothetical protein | 8.69 |
|  | *XC_1400* |  | conserved hypothetical protein | 5.18 |
|  | *XC_3486* |  | hypothetical protein | -1.1 |
|  | *XC_0715* |  | conserved hypothetical protein | -1.09 |
|  | *XC_2561* |  | conserved hypothetical protein | -1.28 |
|  | *XC_2988* |  | conserved hypothetical protein | -1.14 |
|  | *XC_2950* |  | hypothetical protein | 1.79 |
|  | *XC_2621* |  | conserved hypothetical protein | -2.24 |
|  | *XC_2062* |  | conserved hypothetical protein | 5.18 |
