# Supplementary material for: PilG and PilH antagonistically control flagellum-dependent and pili-dependent motility in the phytopathogen Xanthomonas campestris pv. campestris
Source: BMC Microbiol. 2020 Feb 18;20:37. doi: 10.1186/s12866-020-1712-3 (PMC7029496; doi:10.1186/s12866-020-1712-3)
Supplement: Supplementary file 8 — Additional file 8:Table S3. The differential expressed genes of the pilH mutant strain ∆pilH in the rich medium NYGB. [file 12866_2020_1712_MOESM8_ESM.docx]

**Table S3.** The differential expressed genes of the *pilH* mutant strain ∆pilH in the rich medium NYGB.

| Function Category | Gene ID | Name | Annotation | fold change |
| --- | --- | --- | --- | --- |
| Amino acid metabolism | *XC_2377* | *hisH* | glutamine amidotransferase | -1.66 |
|  | *XC_2381* | *hisG* | ATP phosphoribosyltransferase | -1.32 |
|  | *XC_2374* | *hisIE* | phosphoribosyl-ATP pyrophosphohydrolase | -1.07 |
|  | *XC_3156* | *yagT* | xanthine dehydrogenase YagT iron-sulfur-binding subunit | -1.11 |
| Biosynthesis of cofactors, prosthetic groups, carriers | *XC_0983* | *cysG* | sirohydrochlorin ferrochelatase | -1.95 |
|  | *XC_1664* | *gumH* | alpha-1,3-mannosyltransferase | -1.08 |
|  | *XC_1607* | *truB* | tRNA pseudouridine55 synthase | -1.48 |
|  | *XC_1097* | *cobQ* | adenosylcobyric acid synthase | -1.01 |
|  | *XC_1095* | *cobB* | cobyrinic acid a,c-diamide synthase | -1.06 |
|  | *XC_0921* | *lytH* | peptidoglycan LD-endopeptidase LytH | -1.03 |
|  | *XC_0380* | *pcaG* | protocatechuate 3,4-dioxygenase, alpha subunit | -1.02 |
| Cellular Processes | *XC_2235* | *flgC* | flagellar basal-body rod protein | 1.61 |
|  | *XC_2231* | *flgM* | flagellin synthesis | 1.41 |
|  | *XC_2237* | *flaE* | flagellar hook protein | 1.42 |
|  | *XC_2234* | *flgB* | flagellar basal-body rod protein | 1.55 |
|  | *XC_2245* |  | flagellar protein | 1.35 |
|  | *XC_3575* |  | serine protease | -1.54 |
|  | *XC_2244* | *flgL* | flagellar hook-associated protein | 1.33 |
|  | *XC_2240* | *flgH* | flagellar L-ring protein precursor | 1.36 |
|  | *XC_2236* | *flgD* | flagellar basal-body rod modification protein | 1.33 |
|  | *XC_2238* | *flgF* | flagellar basal-body rod protein | 1.32 |
|  | *XC_2246* |  | flagellar protein | 1.22 |
|  | *XC_2243* | *flgK* | flagellar hook-associated protein | 1.25 |
|  | *XC_2298* | *motB* | chemotaxis protein | 1.29 |
|  | *XC_2260* | *fliF* | flagellar M-ring protein | 1.26 |
|  | *XC_2239* | *flgG* | flagellar basal-body rod protein | 1.3 |
|  | *XC_2280* | *flhG* | flagellar biosynthesis protein | 1.32 |
|  | *XC_2279* | *flhF* | flagellar biosynthesis protein | 1.25 |
|  | *XC_2267* | *fliM* | flagellar motor switch protein | 1.15 |
|  | *XC_2247* | *FliS* | flagellar protein | 1.07 |
|  | *XC_2264* | *fliJ* | flagellar protein | 1.24 |
|  | *XC_2266* | *fliL* | flagellar protein | 1.06 |
|  | *XC_2278* | *flhA* | flagellar biosynthesis protein | 1.17 |
|  | *XC_2277* | *flhB* | flagellar biosynthetic protein | 1.28 |
|  | *XC_2272* | *fliQ* | flagellar biosynthetic protein | 1.38 |
|  | *XC_0557* |  | transmembrane sensor | -1.71 |
|  | *XC_2259* | *fliE* | flagellar hook-basal body complex protein | 1.13 |
|  | *XC_2862* |  | fimbrial chaperone protein | 1.12 |
| Energy and carbon metabolism | *XC_1452* |  | formate dehydrogenase related protein | -1.3 |
|  | *XC_0125* |  | pectinesterase | -1.45 |
|  | *XC_3214* | *prpC* | 2-methylcitrate synthase | -1.13 |
|  | *XC_0982* | *cysK* | cysteine synthase A | -1.34 |
|  | *XC_2169* | *rfbF* | glucose-1-phosphate cytidylyltransferase | -1.47 |
|  | *XC_4315* | *glxK* | glycerate 2-kinase | 1.16 |
|  | *XC_0994* | *cysNC* | bifunctional enzyme CysN/CysC | -1.3 |
|  | *XC_0990* | *cysH* | phosphoadenosine phosphosulfate reductase | -1.22 |
|  | *XC_0839* | *ilvM* | acetolactate synthase II small subunit | -1.49 |
| Fatty acid and phospholipid meatbolism | *XC_2191* |  | fatty acid alpha hydroxylase | -1.52 |
|  | *XC_0233* | *ubiJ* | ubiquinone biosynthesis protein | -1.1 |
| Mobile genetic elements | *XC_2007* |  | transposase | 8.27 |
|  | *XC_0412* |  | putative transposase | 2.94 |
|  | *XC_0681* |  | ISxac3 transposase | -1.3 |
|  | *XC_4280* |  | IS1481 transposase | 4.64 |
|  | *XC_2011* |  | IS1477 transposase | -1.6 |
|  | *XC_3804* |  | ISxac3 transposase | 2.34 |
|  | *XC_2623* |  | IS1477 transposase | -1.6 |
| Regulatory functions | *XC_3796* |  | transcriptional regulator lysR family | -1 |
|  | *XC_0245* |  | transcriptional regulator araC family | -1.04 |
|  | *XC_4254* | *slyA* | MarR family transcriptional regulator | -1.5 |
|  | *XC_2157* | *nemR* | TetR/AcrR family transcriptional regulator | 5.17 |
|  | *XC_1766* |  | transcriptional regulator | 1.46 |
|  | *XC_2841* |  | transcriptional regulator | -1.88 |
| Signal transduction | *XC_2314* | *tsr* | methyl-accepting chemotaxis protein I, serine sensor receptor | 2.12 |
|  | *XC_1186* | *pilJ* | twitching motility protein | -1.56 |
|  | *XC_2282* | *cheY* | chemotaxis protein | 1.45 |
|  | *XC_2284* | *CheA* | chemotaxis family, sensor kinase | 1.42 |
|  | *XC_2302* | *cheY* | chemotaxis protein | 1.34 |
|  | *XC_2303* | *cheA* | chemotaxis family, sensor kinase | 1.28 |
|  | *XC_1190* | *chpC* | chemosensory pili system protein | -1.64 |
|  | *XC_1187* | *chpA* | chemosensory pili system protein | -1.36 |
|  | *XC_2223* | *mcp* | chemotaxis protein | 1.26 |
|  | *XC_2321* | *cheR* | chemotaxis protein methyltransferase | 1.43 |
|  | *XC_2300* | *cheW* | chemotaxis protein | -1.46 |
|  | *XC_2793* |  | sensor histidine kinase | 1.75 |
|  | *XC_0638* | *tsr* | chemotaxis protein | 1.24 |
|  | *XC_1188* | *chpB* | chemosensory pili system protein | -1.5 |
|  | *XC_2283* | *cheZ* | chemotaxis protein | 1.23 |
|  | *XC_2318* | *cheW* | chemotaxis protein | 1.28 |
|  | *XC_1413* | *mcp* | chemotaxis protein | 1.39 |
|  | *XC_2311* | *tsr* | chemotaxis protein | 1.25 |
|  | *XC_2163* | *cheB* | protein-glutamate methylesterase | -1.29 |
|  | *XC_2320* | *tsr* | chemotaxis protein | 1.11 |
|  | *XC_1185* | *pilI* | twitching motility protein | -1.46 |
|  | *XC_1414* | *cheA* | chemotaxis family, sensor kinase | 1.21 |
|  | *XC_2306* |  | chemotaxis protein | 1.29 |
|  | *XC_2281* | *fliA* | RNA polymerase sigma factor | 1.09 |
|  | *XC_1801* | *mcp* | chemotaxis protein | 1.07 |
|  | *XC_2297* | *motA* | chemotaxis protein | 1.21 |
|  | *XC_2307* |  | chemotaxis protein | 1.26 |
|  | *XC_2313* |  | chemotaxis protein | 1.04 |
|  | *XC_2304* | *tsr* | chemotaxis protein | 1.06 |
|  | *XC_1410* | *cheR* | chemotaxis protein methyltransferase | 1.04 |
|  | *XC_2309* |  | chemotaxis protein | 1.09 |
| Transporters | *XC_1341* | *fiu* | TonB-dependent receptor | -1.24 |
|  | *XC_0925* |  | iron complex outermembrane recepter protein | -1.09 |
|  | *XC_3700* | *metI* | ABC transporter permease | -1.05 |
|  | *XC_3459* |  | NitT/TauT family transport system permease protein | -1.31 |
|  | *XC_3458* |  | NitT/TauT family transport system ATP-binding protein | -1.21 |
| Undefined category | *XC_2857* |  | protein U | 1.05 |
|  | *XC_2188* |  | glutathione-dependent formaldehyde dehydrogenase | -1.64 |
|  | *XC_2192* |  | glucose 1-dehydrogenase homolog | -1.55 |
|  | *XC_2324* |  | c-di-GMP phosphodiesterase A | 1.25 |
|  | *XC_3170* |  | oxidoreductase | -1.57 |
|  | *XC_3766* |  | oxidoreductase | -1.5 |
|  | *XC_4257* |  | outer membrane efflux protein | -1.36 |
|  | *XC_1201* |  | RebB protein | 1.08 |
|  | *XC_0516* |  | CDP-diacylglycerol-glycerol-3-phosphate 3-phosphatidyltransferase-related protein | -2.06 |
|  | *XC_2552* |  | glycosyltransferase | -1.78 |
|  | *XC_0345* |  | xanthomonadin biosynthesis related protein 1 | 1.53 |
|  | *XC_1345* | *sufD* | ABC transporter permease | -1.19 |
|  | *XC_2785* |  | helicase | 1.44 |
| hypothetical protein | *XC_3761* |  | conserved hypothetical protein | -2.29 |
|  | *XC_2190* |  | conserved hypothetical protein | -2.22 |
|  | *XC_2317* |  | conserved hypothetical protein | 1.66 |
|  | *XC_2830* |  | conserved hypothetical protein | 1.69 |
|  | *XC_2459* |  | conserved hypothetical protein | 1.63 |
|  | *XC_2164* |  | conserved hypothetical protein | -1.98 |
|  | *XC_2786* |  | conserved hypothetical protein | 1.48 |
|  | *XC_2319* |  | conserved hypothetical protein | 1.36 |
|  | *XC_2301* |  | conserved hypothetical protein | 1.32 |
|  | *XC_2050* |  | conserved hypothetical protein | 2.81 |
|  | *XC_3754* |  | conserved hypothetical protein | -1.87 |
|  | *XC_4264* |  | conserved hypothetical protein | -1.83 |
|  | *XC_4035* |  | conserved hypothetical protein | 1.67 |
|  | *XC_3753* |  | conserved hypothetical protein | -1.69 |
|  | *XC_4034* |  | conserved hypothetical protein | 1.49 |
|  | *XC_2787* |  | conserved hypothetical protein | 1.58 |
|  | *XC_2312* |  | conserved hypothetical protein | 1.36 |
|  | *XC_2788* |  | conserved hypothetical protein | 1.26 |
|  | *XC_0251* |  | conserved hypothetical protein | 1.43 |
|  | *XC_2062* |  | conserved hypothetical protein | 6.61 |
|  | *XC_3755* |  | conserved hypothetical protein | -1.85 |
|  | *XC_2353* |  | conserved hypothetical protein | -3.6 |
|  | *XC_2189* |  | conserved hypothetical protein | -1.46 |
|  | *XC_1340* |  | conserved hypothetical protein | -1.78 |
|  | *XC_2230* |  | conserved hypothetical protein | 1.03 |
|  | *XC_2586* |  | conserved hypothetical protein | -1.67 |
|  | *XC_3128* |  | conserved hypothetical protein | -1.41 |
|  | *XC_2861* |  | conserved hypothetical protein | 1.48 |
|  | *XC_3297* |  | conserved hypothetical protein | -2.16 |
|  | *XC_1202* |  | conserved hypothetical protein | 1.23 |
|  | *XC_3756* |  | conserved hypothetical protein | -1.39 |
|  | *XC_0151* |  | conserved hypothetical protein | -1.45 |
|  | *XC_0362* |  | conserved hypothetical protein | 1.11 |
|  | *XC_0430* |  | conserved hypothetical protein | -1.51 |
|  | *XC_0260* |  | conserved hypothetical protein | -1.7 |
|  | *XC_1420* |  | hypothetical protein | 1.17 |
|  | *XC_3525* |  | conserved hypothetical protein | -1.36 |
|  | *XC_2168* |  | conserved hypothetical protein | -1.24 |
|  | *XC_0262* |  | conserved hypothetical protein | -1.84 |
|  | *XC_1045* |  | conserved hypothetical protein | 1.09 |
|  | *XC_2249* |  | conserved hypothetical protein | 1.14 |
|  | *XC_0075* |  | conserved hypothetical protein | 1.48 |
|  | *XC_0071* |  | conserved hypothetical protein | -1.77 |
|  | *XC_2921* |  | conserved hypothetical protein | -1.09 |
|  | *XC_0140* |  | conserved hypothetical protein | -2.13 |
|  | *XC_1339* |  | conserved hypothetical protein | -1.58 |
|  | *XC_2056* |  | conserved hypothetical protein | 1.15 |
|  | *XC_2997* |  | conserved hypothetical protein | -1.36 |
|  | *XC_3893* |  | conserved hypothetical protein | -1.8 |
|  | *XC_1710* |  | conserved hypothetical protein | 1.02 |
|  | *XC_0441* |  | conserved hypothetical protein | -1.02 |
|  | *XC_2953* |  | conserved hypothetical protein | -1.26 |
|  | *XC_2038* |  | conserved hypothetical protein | 1.78 |
|  | *XC_1852* |  | conserved hypothetical protein | -1.09 |
|  | *XC_2185* |  | conserved hypothetical protein | -1.4 |
|  | *XC_1337* |  | conserved hypothetical protein | -1.17 |
|  | *XC_2382* |  | conserved hypothetical protein | -1.72 |
|  | *XC_3764* |  | conserved hypothetical protein | -1.24 |
|  | *XC_3863* |  | conserved hypothetical protein | -1.43 |
|  | *XC_0230* |  | conserved hypothetical protein | -1.2 |
|  | *XC_3784* |  | conserved hypothetical protein | -1.38 |
|  | *XC_0661* |  | conserved hypothetical protein | -1.37 |
|  | *XC_3856* |  | conserved hypothetical protein | -1.08 |
|  | *XC_3752* |  | conserved hypothetical protein | -1.04 |
|  | *XC_4265* |  | conserved hypothetical protein | -1.72 |
|  | *XC_3783* |  | conserved hypothetical protein | -1.29 |
|  | *XC_0989* |  | conserved hypothetical protein | -1.52 |
|  | *XC_3963* |  | hypothetical protein | 8.43 |
|  | *XC_0454* |  | conserved hypothetical protein | -1.21 |
|  | *XC_0727* |  | conserved hypothetical protein | -1.01 |
|  | *XC_2036* |  | conserved hypothetical protein | 2.14 |
|  | *XC_1071* |  | conserved hypothetical protein | -1.02 |
|  | *XC_2987* |  | conserved hypothetical protein | -1.48 |
|  | *XC_4116* |  | conserved hypothetical protein | -1.06 |
|  | *XC_4246* |  | conserved hypothetical protein | -1.26 |
|  | *XC_4012* |  | conserved hypothetical protein | -1.1 |
|  | *XC_3464* |  | conserved hypothetical protein | -1.02 |
|  | *XC_4124* |  | conserved hypothetical protein | -1.01 |
|  | *XC_3838* |  | conserved hypothetical protein | -1.26 |
|  | *XC_3519* |  | conserved hypothetical protein | -1.06 |
|  | *XC_0986* |  | conserved hypothetical protein | -1.14 |
|  | *XC_0089* |  | conserved hypothetical protein | -1.11 |
|  | *XC_3940* |  | conserved hypothetical protein | -1.44 |
|  | *XC_2464* |  | conserved hypothetical protein | 1.28 |
