# Supplementary material for: PilG and PilH antagonistically control flagellum-dependent and pili-dependent motility in the phytopathogen Xanthomonas campestris pv. campestris
Source: BMC Microbiol. 2020 Feb 18;20:37. doi: 10.1186/s12866-020-1712-3 (PMC7029496; doi:10.1186/s12866-020-1712-3)
Supplement: Supplementary file 9 — Additional file 9:Table S4. The overlap of differential expressed genes of the pilG mutant strain ∆pilG and the pilH mutant strain ∆pilH in the rich medium NYGB. [file 12866_2020_1712_MOESM9_ESM.docx]

**Table S4.** The overlap of differential expressed genes of the *pilG* mutant strain ∆pilG and the *pilH* mutant strain ∆pilH in the rich medium NYGB.

| Function Category | Gene ID | Name | Annotation | ΔpilG fold change | ΔpilH fold change |
| --- | --- | --- | --- | --- | --- |
| Biosynthesis of cofactors, prosthetic groups, carriers | *XC_1607* | *truB* | tRNA pseudouridine55 synthase | -1.19 | -1.48 |
| Cellular processes | *XC_2245* | *fliC* | flagellar protein | -1.5 | 1.35 |
|  | *XC_2298* | *motB* | chemotaxis protein MotB | -1.94 | 1.29 |
|  | *XC_2264* | *fliJ* | flagellar FliJ protein | -2.75 | 1.24 |
|  | *XC_2231* | *flgM* | flagellar protein | -1.25 | 1.41 |
|  | *XC_2246* | *fliD* | flagellar protein | -1.03 | 1.22 |
| Energy and carbon metabolism | *XC_0839* | *ilvM* | acetolactate synthase II small subunit | -1.9 | -1.49 |
| Mobile genetic elements | *XC_2007* |  | transposase | 2.32 | 8.27 |
|  | *XC_0412* |  | putative transposase | 2.43 | 2.94 |
|  | *XC_3804* |  | ISxac3 transposase | 2.64 | -2.34 |
| Regulatory functions | *XC_2157* | *nemR* | TetR/AcrR family transcriptional regulator | 4.56 | 5.17 |
|  | *XC_4254* | *slyA* | MarR family transcriptional regulator | -1.48 | -1.5 |
| Signal transduction | *XC_2320* | *tsr* | chemotaxis protein | -2.35 | 1.11 |
|  | *XC_2302* | *CheY* | chemotaxis protein | -1.14 | 1.34 |
|  | *XC_2311* | *tsr* | chemotaxis protein | -1.78 | 1.25 |
|  | *XC_2309* | *tsr* | chemotaxis protein | -1.81 | 1.09 |
|  | *XC_2223* | *mcp* | chemotaxis protein | -1.1 | 1.26 |
|  | *XC_1413* | *mcp* | chemotaxis protein | -1.26 | 1.39 |
|  | *XC_0638* | *tsr* | chemotaxis protein | -1.23 | 1.24 |
|  | *XC_2321* | *cheR* | chemotaxis protein | -1.89 | 1.43 |
|  | *XC_2306* |  | chemotaxis protein | -1.7 | 1.29 |
|  | *XC_2318* | *cheW* | chemotaxis protein | -1.94 | 1.28 |
|  | *XC_1410* | *cheR* | chemotaxis protein | -1.69 | 1.04 |
|  | *XC_2303* | *cheA* | chemotaxis protein | -1.14 | 1.28 |
|  | *XC_2314* | *tsr* | chemotaxis protein | -1.02 | 2.12 |
|  | *XC_1414* | *cheA* | chemotaxis protein | -1.07 | 1.21 |
|  | *XC_2313* | *tsr* | chemotaxis protein | -1.28 | 1.04 |
| Transport | *XC_1341* | *fiu* | TonB-dependent receptor | -1.06 | -1.06 |
| Undefined category | *XC_1201* |  | RebB protein | -1.53 | 1.08 |
|  | *XC_2785* |  | helicase | -1.16 | 1.44 |
|  | *XC_0516* |  | CDP-diacylglycerol-glycerol-3-phosphate 3-phosphatidyltransferase-related protein | -1.37 | -2.06 |
| hypothetical protein | *XC_2319* |  | conserved hypothetical protein | -1.82 | 1.36 |
|  | *XC_1202* |  | conserved hypothetical protein | -1.04 | 1.23 |
|  | *XC_2830* |  | conserved hypothetical protein | -1.23 | 1.69 |
|  | *XC_2786* |  | conserved hypothetical protein | -1.11 | 1.48 |
|  | *XC_2301* |  | conserved hypothetical protein | -1.49 | 1.32 |
|  | *XC_2788* |  | conserved hypothetical protein | -1.06 | 1.26 |
|  | *XC_2230* |  | conserved hypothetical protein | -1.29 | 1.03 |
|  | *XC_4034* |  | conserved hypothetical protein | -1.26 | 1.49 |
|  | *XC_0362* |  | conserved hypothetical protein | -1.26 | 1.11 |
|  | *XC_2249* |  | conserved hypothetical protein | -1.08 | 1.14 |
|  | *XC_2353* |  | conserved hypothetical protein | -1.87 | -3.6 |
|  | *XC_0262* |  | conserved hypothetical protein | -3.08 | -1.84 |
|  | *XC_2036* |  | conserved hypothetical protein | 3.07 | 2.14 |
|  | *XC_2050* |  | conserved hypothetical protein | 2.48 | 2.81 |
|  | *XC_3525* |  | conserved hypothetical protein | -1.6 | -1.36 |
|  | *XC_3755* |  | conserved hypothetical protein | -1.1 | -1.85 |
|  | *XC_0661* |  | conserved hypothetical protein | -1.89 | -1.37 |
|  | *XC_1340* |  | conserved hypothetical protein | -1.22 | -1.22 |
|  | *XC_3784* |  | conserved hypothetical protein | -1.67 | -1.38 |
|  | *XC_3893* |  | conserved hypothetical protein | -1.81 | 1.8 |
|  | *XC_3128* |  | conserved hypothetical protein | -1.01 | -1.41 |
|  | *XC_4246* |  | conserved hypothetical protein | -1.66 | -1.26 |
|  | *XC_0233* |  | conserved hypothetical protein | -1.2 | -1.1 |
|  | *XC_1337* |  | conserved hypothetical protein | -1.11 | -1.17 |
|  | *XC_3783* |  | conserved hypothetical protein | -1.15 | -1.29 |
|  | *XC_3297* |  | conserved hypothetical protein | -1.43 | -2.16 |
|  | *XC_4116* |  | conserved hypothetical protein | -1 | -1.06 |
|  | *XC_3940* |  | conserved hypothetical protein | 8.69 | -1.44 |
|  | *XC_2062* |  | conserved hypothetical protein | 5.18 | -6.61 |
