# Supplementary material for: PilG and PilH antagonistically control flagellum-dependent and pili-dependent motility in the phytopathogen Xanthomonas campestris pv. campestris
Source: BMC Microbiol. 2020 Feb 18;20:37. doi: 10.1186/s12866-020-1712-3 (PMC7029496; doi:10.1186/s12866-020-1712-3)
Supplement: Supplementary file 10 — Additional file 10:Table S5. Primers used in this study. [file 12866_2020_1712_MOESM10_ESM.docx]

**Table S5.** Primers used in this study^§^

| Primers | Nucleotide sequence (5′→3′) | The amplified fragment or the utilization |
| --- | --- | --- |
| LpilG-F  LpilG-R | GGGGAATTCTTGCGCATCAGCACCACCTGGCCG  GGGTCTAGATTCAGTCATGCAAATATCCCCGG | 521-bp DNA sequence upstream of *pilG*, used for construction of *pilG* deletion mutant |
| RpilG-F  RpilG-R | GGGTCTAGAGTCAACGCCTGACCAGGGGGAAAGGCAACATGGCTC  GGGAAGCTTACGATGTAGTCACTTGCACCCTG | 327-bp DNA sequence downstream of *pilG*, used for construction of *pilG* deletion mutant |
| CpilG-F  CpilG-R | AGATCCGGATCCATGACTGAAAACATGGCTGCGGGTGG  GGGAAGCTTACGATGTAGTCACTTGCACCCTG | DNA fragment of 729-bp *pilG* coding sequence, us used for complementation of *pilG* deletion mutant |
| LpilH-F  LpilH-R | GGGGAATTCATATTTGCATGACTGAAAACATGGCTGCGGGTG  GGGTCTAGATGGTCAGGCGTTGACGTACGTAC | 425-bp DNA sequence upstream of *pilH*, used for construction of *pilH* deletion mutant |
| RpilH-F  RpilH-R | GGGTCTAGAAACTGGTGCGTTGATGCGTTCTC  GGGAAGCTTAGCTCATCGATGGTCAGCGCGAC | 384-bp DNA sequence downstream of *pilH*, used for construction of *pilH* deletion mutant |
| CpilH-F  CpilH-R | ACAGTTGGATCCATGGCTCGAATCATATTGATCGA  GGGAAGCTTAGCTCATCGATGGTCAGCGCGAC | DNA fragment of 786-bp *pilH* coding sequence, used for complementation of *pilH* deletion mutant |
| LfilM-F  LfliM-R | GGGGGATCCTGGTTCTTCCTCGGCCACAAG  GGGTCTAGAGGGGACGGTGCCTTACTGGGT | 426-bp DNA sequence upstream of *fliM*, used for construction of *fliM* deletion mutant |
| RfliM-F  RfliM-R | GGGTCTAGAATGATCAACTCCGACATCCTC  GGGAAGCTTGTCGTTGATCACCACCACTTC | 294-bp DNA sequence downstream of *fliM*, used for construction of *fliM* deletion mutant |
| CfliM-F  CfliM-R | GGGGGATCCATGAGCGTCAGTGATCTG  GGGAAGCTTAGGTTGCGGATCGGAATG | DNA fragment of 1189-bp *fliM* coding sequence, used for complementation of *fliM* |
| LfilN-F  LfliN-R | CGGGATCCAGATCCACATCACCCTGCCGTACTCGAT  GCTCTAGATTGATCATTTGCTGGAGTCC | 387-bp DNA sequence upstream of *fliN*, used for construction of *fliN* deletion mutant |
| RfliN-F  RfliN-R | GCTCTAGAGCTGCGGTGATCGGCCTGCT  CCAAGCTTAATTGGGCAGGGTGGGTGCA | 367-bp DNA sequence downstream of *fliN*, used for construction of *fliN* deletion mutant |
| CfliN-F  CfliN-R | CGGAATTCGATGATCAACTCCGACATCCTCGACGCCG  CCAAGCTTTCACCGCAGCCTCCGGATCC | DNA fragment of 352-bp *fliN* coding sequence, used for complementation of *fliN* deletion mutant |
| LpilB-F  LpilB-R | CGGAATTCTTGAGGCTGGATTGGGGAGTCCTGAAAG  GCTCTAGATACGAAAGGACAAGCATTTG | 304-bp DNA sequence upstream of *pilB*, used for construction of *PilB* deletion mutant |
| RpilB-F  RpilB-R | GCTCTAGAGCGCCGTTTCTTTTGGTGTAGT  CCAAGCTTAACAAGACCAAGACCGCCGC | 514-bp DNA sequence downstream of *pilB*, used for construction of *pilB* deletion mutant |
| CpilB-F  CpilB-R | CGGAATTCGATGAGCGTTGTGCTAACGGCTAATCTTG  CCAAGCTTTCAGTCCTTGGTCACACGAT | DNA fragment of 1747-bp *PilB* coding sequence, used for complementation of *pilB* deletion mutant |
| LcheA-F  LcheA-R | ACAGTTGGATCCATGTCGAGGTGAGAACTT  ACAGTTTCTAGAGTCTTCGAGCAGATCCCG | 494-bp DNA sequence upstream of *cheA*, used for construction of *cheA* deletion mutant |
| RcheA-F  RcheA-R | ACAGTTTCTAGAGTGATCAAGCCATTGTCC  ACAGTTAAGCTTAGGGCGACATCGAAGTTG | 493-bp DNA sequence downstream of *cheA*, used for construction of *cheA* deletion mutant |
| CcheA-F  CcheA-R | ACAGTTGGATCCCGAACGAATTCATTAACA  ACAGTTAAGCTTCTGGAGTCAGGGAACGAC | DNA fragment of 2124-bp *cheA* coding sequence, used for complementation of *cheA* deletion mutant |
| L2306-F  L2306-R | ACAGTTGAATTCCTGCTCCGAGATTAGTGT  ACAGTTTCTAGATTGCAATCGTGGTCTTGA | 429-bp DNA sequence upstream of *XC_2306*, used for construction of *XC_2306* deletion mutant |
| R2306-F  R2306-R | ACAGTTTCTAGACAAGCCGCTGAACGATGT  ACAGTTAAGCTT ATCATCACCTTGGTCTGC | 492-bp DNA sequence downstream of *XC_2306*, used for construction of *XC_2306* deletion mutant |
| C2306-F  C2306-R | ACAGTTGGATCCCTAGCACATTGAGTGTCA  ACAGTTAAGCTTGTGTCGTCCAGGTACACG | DNA fragment of 685-bp *XC_2306* coding sequence, used for complementation of *XC_2306* deletion mutant |
| LcheY-F  LcheY-R | ACAGTTGGATCCGCCTGTGCCGCATTTCCAAC  ACAGTTTCTAGATGCGGCGTTCTCCACAGA | 514-bp DNA sequence upstream of *cheY*, used for construction of *cheA* deletion mutant |
| RcheY-F  RcheY-R | ACAGTTTCTAGACCACCGTCCAGAAAGTCC  ACAGTTAAGCTTGGCTTCGGGTTCTTCTTC | 516-bp DNA sequence downstream of *cheA*, used for construction of *cheA* deletion mutant |
| CcheY-F  CcheY-R | ACAGTTGGATCCATGGGCGTGAAATCCGAT  ACAGTTAAGCTTTGACAACCTGAAATCTGT | DNA fragment of 449-bp *cheY* coding sequence, used for complementation of *cheY* deletion mutant |
| pilGFlag-F  pilGFlag-R | CGGGATCCGACTACAAAGACCATGACGGTGATTATAAAGATCATGATATCGACTAC  AAAGATGACGACGATAAAATGACTGAAAACATGGCTGC  GGGAAGCTTACGATGTAGTCACTTGCACCCTG | DNA fragment of 795-bp *pilG* coding sequence containing 3×Flag, used for complementation, overproduction of *pilG* |
| pilHFlag-F  pilHFlag-R | CGGGATCCGACTACAAAGACCATGACGGTGATTATAAAGATCATGATATCGACTAC  AAAGATGACGACGATAAAATGGCTCGAATCATATTGATCGA  GGGAAGCTTAGCTCATCGATGGTCAGCGCGAC | DNA fragment of 852-bp *pilG* coding sequence containing 3×Flag, used for complementation, overproduction of *pilG* |
| 2320-F  2320-R | GTGTCAATGCAATGGATCAA  TCACACCGTCGATGTCTTTG | 304-bp DNA fragment spans nucleotides 1 to 304 bp of the *XC_2320*, used for RT-PCR. |
| 2302-F  2302-R | ATGAGCGCACGTATCTTGGT  TTCGGATTTCTTGTCGGCTG | 279-bp DNA fragment spans nucleotides 1 to 279 bp of the *XC_2302*, used for RT-PCR |
| 2311-F  2311-R | ATGGTGAATGCCAACTACGT  TTGAGCACCTTGGTCTCTGC | 215-bp DNA fragment spans nucleotides 1 to 215 bp of the *XC_2311*, used for RT-PCR |
| 2309-F  2309-R | TGAACAAGTTCAACGATTG  AAGAAATACGCATAGCGCTC | 260-bp DNA fragment spans nucleotides 1 to 260 bp of the *XC_2309*, used for RT-PCR |
| 1413-F  1413-R | GCAACTTCGATGTCGCCCTG  TGGCGACCATGCGGTTGATG | 219-bp DNA fragment spans nucleotides 80 to 298 bp of the *XC_1413*, used for RT-PCR |
| 0638-F  0638-R | ATGCAGCACTACGCCGACAA  CGGTCTTGATATCGCGGTAG | 229-bp DNA fragment spans nucleotides 1 to 229 bp of the *XC_0638*, used for RT-PCR |
| 2321-F  2321-R | ATGGATATGACGACGACCTC  CATTCTTCATCGTCGTTGCC | 233-bp DNA fragment spans nucleotides 1 to 233 bp of the *XC_2321*, used for RT-PCR |
| 2306-F  2306-R | ATGACGTTCAAGACCACGAT  GTAGATGTAATCCACGCCGC | 309-bp DNA fragment spans nucleotides 1 to 309 bp of the *XC_2306*, used for RT-PCR |
| 1410-F  1410-R | ATGCCGACCTTGTCTGAGCC  CGCGGAAGAAATAGGTTTCG | 271-bp DNA fragment spans nucleotides 1 to 271 bp of the *XC_1410*, used for RT-PCR |
| 2303-F  2303-R | ATGAGCATGGACCTGCAACG  CCGCATTGGGTTCGAGTTGG | 262-bp DNA fragment spans nucleotides 1 to 262 bp of the *XC_2303*, used for RT-PCR |
| 2314-F  2314-R | ATGCAGCACCTGCAGGACATGACC  CTTCGTCGGATTTGGAGGCG | 277-bp DNA fragment spans nucleotides 1 to 277 bp of the *XC_2314*, used for RT-PCR |
| 1414-F  1414-R | GGACATGAACCAGCTGATGC  CATGGATGTGGTCGCAGCAC | 290-bp DNA fragment spans nucleotides 3 to 292 bp of the *XC_1414*, used for RT-PCR |
| 2301-F  2301-R | ATGAGCACAGTGATATTGGG  GGCCGCATCTCTAAAAGTGT | 231-bp DNA fragment spans nucleotides 1 to 231 bp of the *XC_2301*, used for RT-PCR |
| 2785-F  2785-R | AGCTTCGCTTCTGGAAAGGA  TTTGTGTCGGTTGGTGTCCC | 310-bp DNA fragment spans nucleotides 1 to 310 bp of the *XC_2785*, used for RT-PCR |
| 2230-F  2230-R | ATGAACGTGAACGATTCCCT  ATACGCTGGACTGGCCATTG | 301-bp DNA fragment spans nucleotides 1 to 301 bp of the *XC_2230*, used for RT-PCR |
| 2245-F  2245-R | ATGGCACAGGTAATCAACACC  TGTTGCCGATTTCGACCATC | 262-bp DNA fragment spans nucleotides 1 to 262 bp of the *XC_2245*, used for RT-PCR |
| 2264-F  2264-R | ATGATGCAGTCCAAACGAAT  ATTGCTCCAGTACCTGTTTT | 328-bp DNA fragment spans nucleotides 1 to 328 bp of the *XC_2264*, used for RT-PCR |
| 1201-F  1201-R | ATGGCTTTTCCAACCGCAGT  TTGCGCGACCTTCTCGACGT | 258-bp DNA fragment spans nucleotides 1 to 258 bp of the *XC_1201*, used for RT-PCR |
| 16SF  16SR | GCCTAACACATGCAAGTCGAACGGC  AATATTCCCCACTGCTGCCTCCCG | 325-bp DNA fragment of the 16S rDNA sequence, used for RT-PCR. |
| PpilG-F  PpilG-R | AGATCCGGATCCATGACTGAAAACATGGCTGCGGGTGG  GGGCTCGAGACGATGTAGTCACTTGCACCCTG | DNA fragment of 729-bp *pilG* coding sequence, used for overexpression and bacterial two-hybrid of PilG |
| PpilH-F  PpilH-R | ACAGTTGGATCCATGGCTCGAATCATATTGATCGA  GGGCTCGAGAGCTCATCGATGGTCAGCGCGAC | DNA fragment of 786-bp *pilH* coding sequence, used for overexpression and bacterial two-hybrid of PilH |
| pBTfliN-F  pBTfliN-R | CGGAATTCGATGATCAACTCCGACATCCTCGACGCCG  CCCTCGAGTCACCGCAGCCTCCGGATCC | DNA fragment of 382-bp *fliN* coding sequence, used for overexpression and bacterial two-hybrid |
| pBTfliM-F  pBTfliM-R | CGGGATCCATGAGCGTCAGTGATCTGCTTTCC  GGGCTCGAGTCATTTGCTGGAGTCCTGGGAGG | DNA fragment of 1032-bp *fliM* coding sequence, used for bacterial two-hybrid |
| P3619-F  P3619-R | CGGGATCCGTGAGTCATTTCGTGCACCCCA  GGGCTCGAGTCACCGGGCCGCATTGGCAA | DNA fragment of 942-bp *XC_3619* coding sequence, used for bacterial two-hybrid |
| BpilN-F  BpliN-R | CGGGATCCATGGCAAGAATCAATCTATTGC  GGGCTCGAGTCATGACGCCGCCCCCTCCT | DNA fragment of 792-bp *pilN* coding sequence, used for bacterial two-hybrid |
| PcheA_2284_-F  PcheA_2284_-R | CGGGATCCATGAGTGCTGTTCCAGACGA  GGGCTCGAGTCAATGATCGCTGGAACGCA | DNA fragment of 1668-bp *CheA_2284_* coding sequence, used for and bacterial two-hybrid |
| PpilU-F  PpilU-R | CGGGATCCATGAGCACCATCGACTTCAC  GGGCTCGAGTTACCGAACCTCGGCAATCT | DNA fragment of 1143-bp *pilU* coding sequence, used for and bacterial two-hybrid |
| PpilR-F  PpilR-R | CGGGATCCGTGCGCAACATGAACGAAACGAAAAGTGC  GGGCTCGAGCTACTCCATCCCCAATTTCT | DNA fragment of 1416-bp *pilR* coding sequence, used for bacterial two-hybrid |
| P1163-F  P1163-R | CGGGATCCATGTCCAAGAAGAAGAACGCAGC  CCGCTCGAGTTACTGCAGCAGCGAGCGCA | DNA fragment of 564-bp *XC_1163* coding sequence, used for and bacterial two-hybrid |
| PphoP-F  PphoP-R | CGGGATCCATGCGTATCCTTTTGGTCGA  CCGCTCGAGCTGTGCTGACTCAGCCTTCC | DNA fragment of 711-bp *phoP* coding sequence, used for and bacterial two-hybrid |
| P1378-F  P1378-R | CGGGATCCATGGCCCGCGGCATCAACAA  CCGCTCGAGTTAGAACGGGATATCGTCGT | DNA fragment of 536-bp *XC_1378* coding sequence, used for and bacterial two-hybrid |
| P3262-F  P3262-R | CGGGATCCATGAATAAAACCGAATTGAT  CCGCTCGAGTTAGTTTACTGCATCCTTCA | DNA fragment of 290-bp *XC_3262* coding sequence, used for bacterial two-hybrid |
| P3597-F  P3597-R | CGGGATCCATGTCGATCGATCTCACCGG  CCGCTCGAGTCACGCAGCCTTGGCTTTCT | DNA fragment of 485-bp *XC_3597* coding sequence, used for and bacterial two-hybrid |
| BcolS-F  BcolS-R | CGGGATCCATGAACCGCAATATCGACGC  CCGCTCGAGCGCTTCATGATTCCAGGACT | DNA fragment of 1267-bp *colS* coding sequence, used for bacterial two-hybrid |
| PpilI-F  PpilI-R | CGGGATCCTTGATGCGTTCTCCATTCGA  CCGCTCGAGACCTCGATCTGTATGACGTG | DNA fragment of 584-bp *pilI* coding sequence, used for bacterial two-hybrid |
| P1355-F  P1355-R | CGGGATCCATGGCAAAAACCGCCGCTAAGAAGG  CCGCTCGAGAGACGTGGCAAACGCGCAAAA | DNA fragment of 507-bp *XC_1355* coding sequence, used for and bacterial two-hybrid |
| LpilI-F  LpilI-R | CGGGATCCTCCGAGCAATATCTGACCAA  GCTCTAGAAACGCACCAGTTGTTTGATG | 469-bp DNA sequence upstream of *pilI* used for construction of *pilI* deletion mutant |
| RpilI-F  RpilI-R | GCTCTAGAGCCGCGACCCCGCCACGTCAT  CCAAGCTTGCGGCCCTTGGTCTCCTTGA | 316-bp DNA sequence downstream of *pilI*, used for construction of *pilI* deletion mutant |
| CpilI-F  CpilI-R | CGGGATCCATGCGTTCTCCATTCGACATTCT  CCAAGCTTGCGGCCCTTGGTCTCCTTGA | DNA fragment of 550-bp *pilI* coding sequence, used for complementation of *pilI* deletion mutant |
| LcolS-F  LcolS-R | CGCGGATCCAACTGCCCGACTCGGATT  TGCTCTAGAGGTTCATCACGTCCTCCA | 307-bp DNA sequence upstream of *colS* used for construction of *colS* deletion mutant |
| RcolS-F  RcolS-R | ACATCTAGAAGGGCCTTACCGGCAATGCTTCAC  ACAAAGCTTAGGATGACCGCGTGGTGGCCTATT | 480-bp DNA sequence downstream of *colS*, used for construction of *colS* deletion mutant |
| CcolS-F  CcolS-R | CGGGATCCATGCCTGAGAGCTCCAGCGG  ACAAAGCTTTTAGCGATGGAACGCCAGTG | DNA fragment of 1352-bp *colS* coding sequence, used for complementation of *colS* deletion mutant |
